# The Motion of Body Center of Mass During Walking: A Review Oriented to Clinical Applications

**DOI:** 10.3389/fneur.2019.00999

**Published:** 2019-09-20

**Authors:** Luigi Tesio, Viviana Rota

**Affiliations:** ^1^Department of Biomedical Sciences for Health, Università degli Studi, Milan, Italy; ^2^Department of Neurorehabilitation Sciences, Istituto Auxologico Italiano, IRCCS, Milan, Italy

**Keywords:** walking, body center of mass, pathological gaits, system approach, gait rehabilitation

## Abstract

Human walking is usually conceived as the cyclic rotation of the limbs. The goal of lower-limb movements, however, is the forward translation of the body system, which can be mechanically represented by its center of mass (CoM). Lower limbs act as struts of an inverted pendulum, allowing minimization of muscle work, from infancy to old age. The plantar flexors of the trailing limbs have been identified as the main engines of CoM propulsion. Motion of the CoM can be investigated through refined techniques, but research has been focused on the fields of human and animal physiology rather than clinical medicine. Alterations in CoM motion could reveal motor impairments that are not detectable by clinical observation. The study of the three-dimensional trajectory of the CoM motion represents a clinical frontier. After adjusting for displacement due to the average forward speed, the trajectory assumes a figure-eight shape (dubbed the “bow-tie”) with a perimeter about 18 cm long. Its lateral size decreases with walking velocity, thus ensuring dynamic stability. Lateral redirection appears as a critical phase of the step, requiring precise muscle sequencing. The shape and size of the “bow-tie” as functions of dynamically equivalent velocities do not change from child to adulthood, despite anatomical growth. The trajectory of the CoM thus appears to be a promising summary index of both balance and the neural maturation of walking. In asymmetric gaits, the affected lower limb avoids muscle work by pivoting almost passively, but extra work is required from the unaffected side during the next step, in order to keep the body system in motion. Generally, the average work to transport the CoM across a stride remains normal. In more demanding conditions, such as walking faster or uphill, the affected limb can actually provide more work; however, the unaffected limb also provides more work and asymmetry between the steps persists. This learned or acquired asymmetry is a formerly unsuspected challenge to rehabilitation attempts to restore symmetry. Techniques of selective loading of the affected side, which include constraining the motion of the unaffected limb or forcing the use of the affected limb on split-belt treadmills which impose a different velocity and power to either limb, are now under scrutiny.

## Walking: Moving the Body Segments in Order to Translate the Body System

A huge amount of research, a true odyssey (1), has been dedicated to the physiology of human walking. Although increased physiological knowledge has deepened our understanding, clinical science is still waiting for a consensus on the best strategy for diagnosis and treatment of many walking impairments. Human walking includes motion of virtually all body segments, meaning that alteration of the motion of any one segment induces adaptive movements across the whole body. In some cases, local malfunctioning is responsible for the impairment, while in others, such as individuals presenting with balance deficits or paresis due to diffused lesions of the central nervous system, it may be difficult to interpret the numerous concurrent alterations and identify critical targets for clinical observation. These alterations may be causes or effects of the underlying impairment and might, therefore, represent either additional impairments or useful adaptive mechanisms. This review is focused on the study of walking seen as the translation of the body system as a whole, represented by its center of mass (CoM) and aims at demonstrating that abstracting from segmental motions may help clinicians interpreting and possibly, treating the segmental impairments. Wide fields of research relating to CoM motion, such as studies on running or on the various forms of gait of legged animals, such as walking, running, trotting, canter, galloping, hopping, and so on, were treated tangentially to the main topic or disregarded. Studies of metabolic energy expenditure during walking, which can be considered as a form of system approach, were only treated marginally when the results were closely related to the CoM motion. Only studies of steady-state human walking on level ground in a straight direction, both in health and disease, were considered. Studies of the physiology of gait initiation (2) and termination (3) were ignored, along with a number of others including those of walking up- and downhill (4), along curved trajectories (5), across expected and unexpected obstacles (6), and in hypo- and hyper-gravity (7) among many others. The general principles of the physiology of walking in Man and, in general, in legged animals, are extensively covered in excellent, comprehensive textbooks (8–10).

### The Body System as a Whole: The Concept of Center of Mass

The body system as a whole may be represented, from a mechanical standpoint, by its CoM. The CoM of a distribution of mass is the unique point in space whose linear acceleration is determined only by the total external force acting on the system, without effects due to internal forces (11). When applied to the CoM, such force causes a linear acceleration without angular acceleration. One may also describe the CoM as the unique point which invariably lies in planes dividing the body into two parts, sharing the same moment of inertia (see Note S1 for a mathematical description). The CoM usually moves within the body when body segments are displaced with respect to each other. This “center” may even lie outside the body mass. In a donut, the CoM lies in the hole. By keeping the CoM outside the body and below the bar by arching his body, Dick Fosbury was able to unexpectedly win the high-jump gold medal in the 1968 Olympic games in Mexico (12). These familiar examples show that, albeit virtual, the CoM is far from being imaginary. Some help in grasping the CoM concept in the study of walking may come from imagining the displacement of a point on a screen tracking a GPS transmitter embedded in the belt of a walking subject (although, the real CoM is free to move outside the belt). Whichever examples and metaphors are suggested, it remains true that thinking of the CoM, a virtual point, requires abstraction from morphology and therefore, from anatomy, which is the reason why it is often neglected in clinical studies.

### Highlighting the Two Opposing Viewpoints

Two distinct viewpoints still characterize the study of human legged animals' walking (13).

#### Walking as a Sequence of Joint Rotations

The earlier, yet still dominant, viewpoint considers walking to be a series of cyclic rotations of the limbs and trunk, referred to as the “segmental” approach or viewpoint from here on. To illustrate this concept, one can imagine a man walking on a treadmill; the cyclic nature of the joint rotations is apparent. The beginning of the cycle is a matter of convention; usually, but not exclusively, this is considered as the instant of heel strike. The series of events that takes place between subsequent heel strikes of opposite sides of the body is called a step. Two subsequent steps make a stride.

#### Walking as the Translation of the Body System as a Whole

The less widely accepted viewpoint highlights that walking has the primary goal of achieving forward translation of the body system as a whole (locomotion), although this results from the interplay between gravity and the coordinated and cyclic contraction of numerous muscles. In this view, the body system as a whole can be represented by its CoM. This will be referred to as the “system” viewpoint or approach from here on.

### The CoM Motion: The Cinderella of Gait Analysis

The umbrella term “gait analysis” describes the synchronous recording of many segmental variables, which has become accessible to clinical settings in recent decades, due to the rapid progression of technology [for kinematics, see (14)]. However, the CoM motion is not yet considered in routine gait analysis. This can be a reason for its limited success in clinical practice, as it will be discussed later in this Review.

## Modeling the Walking Phenomenon

Anthropometric studies have demonstrated that the CoM of quietly standing humans lies approximately a few centimeters in front of the lumbosacral joint, about 57% of the body height from the feet plants, in both children and adults (15). Sophisticated modeling has allowed the estimation of its location in various postures and during various motor tasks; again, in children (15) and in adults (16, 17). Tracking the CoM during walking, however, is much more difficult.

### The CoM Motion as the Oscillation of an Inverted Pendulum: An Old Intuition

How to measure the motion parameters of the invisible CoM during walking has been a challenge for generations of researchers. A first approximation was reducing the body motion to the visible motion of the sacrum. This approximation is rather rough. In fact, the movement of the CoM is affected by the movement of individual body segments, while the sacral markers are not (18). However, this simplification had the advantage of reinforcing the very old intuition that the CoM moves up and down and accelerates and decelerates forward during each step, like an inverted pendulum, of which a mechanic metronome is perhaps the most familiar example.

**Figure 1:**
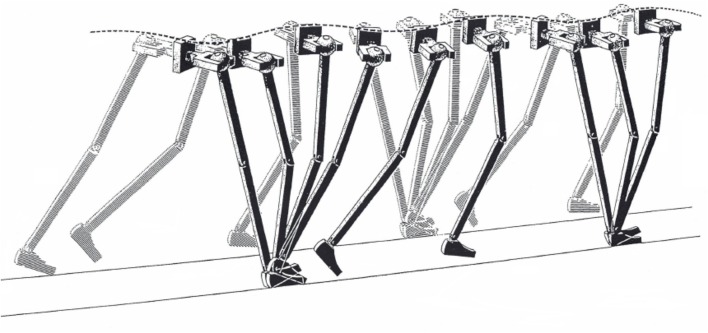
Illustration of the “inverted pendulum” model of walking. Some of the sagittal, frontal, and horizontal rotations of the joints are seen as major determinants of gait, as long as they usefully smooth the trajectory of the body center of mass. Taken from Saunders et al. (19), used with permission.

A famous clinical article has suggested that smoothing the CoM oscillations is the aim of some of the segmental motions of the lower limbs, such as pelvic tilt and knee flexion, named the six “major determinants” of gait (19) (Figure 1).

The authors suggested that these six determinants deserved special attention in clinical observation. Unfortunately, measuring energy changes and displacements of the human CoM proved to be considerably more challenging than intuitively capturing the pendulum analogy.

### Modern Methods for Analyzing the CoM Motion

Nowadays, numerous and valid methods exist to observe and measure the CoM motion. In this review, articles based on the “sacral marker” simplification will be overlooked. Articles will be considered, based on the analysis of ground reaction forces (the so-called “double integration” or Newtonian method) or on kinematic analysis of body segments (usually, through optoelectronic “capturing” of retroreflective skin markers) as per anthropometric modeling. Both methods provide reliable and very similar results with respect to the CoM motion. The corresponding technicalities and criticisms are summarized in the following paragraphs and in Note S2. The overground walking mechanism can be reproduced and measured more easily on a treadmill. The treadmill imposes a known and constant average velocity, thus enabling the investigator to record several reproducible steps in a matter of seconds. Experimental sessions can be very short, which is advantageous for the examination of children or impaired adults. The treadmill also imposes reproducibility of velocity across time points. Furthermore, these devices can be mounted on force sensors, thus providing a virtually unlimited distance with embedded force sensors in a limited space. Although the two walking modalities, in theory, are mechanically equivalent, they present with some behavioral differences. The treadmill approach has both limitations and advantages which are discussed in a dedicated paragraph and in Note S3. Nevertheless, given their substantial similarity, results based both of ground and treadmill walking will be presented here. Some consideration will be given here to walking on treadmills made of two independent belts running at different velocities (split- or dual-belt treadmills). These devices are of interest because they induce an artificial form of limping, and might offer possible therapeutic approaches to help unilaterally impaired subjects recover symmetric motion of the CoM (see further paragraphs).

### The Three-Dimensional Path of the Center of Mass: Seeing the Invisible

The 3D trajectory of the CoM is among the most recent outcomes of an old line of research. The only abstraction required to the clinician is subtracting the average forward velocity of the body: stated otherwise, one can imagine observing a subject walking on a treadmill, with zero average velocity with respect to the ground. The subject's trunk will show rhythmic back-forth, and left-right oscillations, with no average forward displacement. This approach, adopted here, will not require delving into the underlying principles of physics and engineering. Also, it might better fit the clinical assessment, still based mostly on visual observation of gait impairments.

### From 2D to 3D Analysis. Considering the Lateral Motion

The pendulum-like CoM motion was usually analyzed in the sagittal plane although, in principle, the same technology could well be applied to the frontal and horizontal planes. On the other hand, most of the work to move the body system and its segments, and its largest displacements, can be observed in the sagittal plane (20) so that most of the conclusions on work production do not change remarkably when the other planes are also considered. The lateral displacement of the CoM, however, may be of crucial importance from a clinical standpoint, because lateral stability is challenged in many pathologic conditions that are caused by neurologic or orthopedic impairments. At each step, the CoM oscillates laterally toward the supporting leg, then swings toward the opposite leg during the next step, producing an inverted pendulum-like mechanism in the frontal plane, too. The composite motion of the sagittal and frontal pendulum during a stride follows a curved path around the line of progression. If advancement due to the average forward velocity is subtracted (instantaneous velocity still undergoes periodic changes, of course) the CoM path assumes a closed figure-eight shape, upwardly concave in the frontal plane, with an overall length of about 18 cm (**Figure 3**).

A systematic study of the 3D path of the CoM in healthy adults at various walking velocities, based on the double-integration method (see above, and Note S2), was first published in 2010 (21). This elegant representation of CoM motion was dubbed the “bow-tie,” and planar projections of this figure were first demonstrated in two studies: one related to the 3D motion of the CoM in healthy adults, patients with unilateral hip-joint replacement and patients with post-stroke hemiparesis (22); the other analyzed the CoM path in the frontal plane only, in children with various forms of cerebral palsy and in healthy controls (23). The bow-tie shape has since been confirmed and mathematically modeled in an independent study (24).

### Linking the CoM Path to Walking Velocity

**Figure 2:**
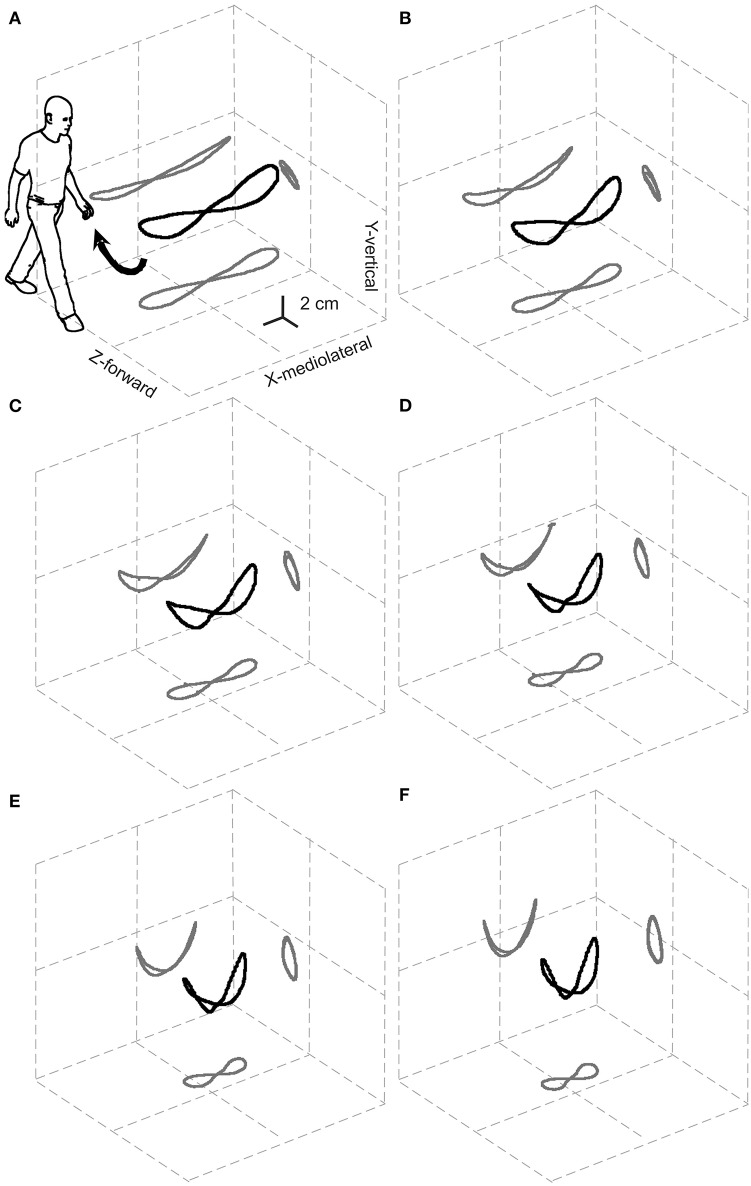
Relationships between the 3D path of the center of mass (CoM) during one stride and the walking speed. The black and gray curves represent the 3D path of the CoM and their planar projections, respectively, once the displacement due to the forward velocity has been subtracted. Results are grand-averaged across 18 subjects. The resulting elegant figure is dubbed the “bow-tie.” The range of speeds tested, illustrated in **(A–F)**, was partitioned into increasing sextiles from 0.01 to 0.27 a-dimensional, leg length adjusted Froude units (0.3–1.4 m s^−1^). Taken from Tesio et al. (21), used with permission.

Most velocity-related changes in the total length of the curved bow-tie path occur due to the shortening of its lateral oscillations (Figure 2).

Furthermore, the path length shows a U-shaped relationship to the average forward velocity with a minimum of around 1.3 m s^−1^, which is very close to the optimum speed with regards to the minimization of metabolic energy expenditure and maximization of passive exchange between kinetic and gravitational potential energy of the CoM (21).

Notably, the upward concavity of the bow-tie increases with increasing velocity, so that lateral oscillations of the CoM, unlike the total path length, are monotonically restricted from about 10 cm at 0.3 m s^−1^ to about 5 cm at 1.4 m s^−1^. This might explain why ataxic subjects are, paradoxically, more stable at faster walking speeds. Although this phenomenon is well-known by clinicians, it is documented only anecdotally (25, 26). This has been interpreted as a favorable shift toward a more automatic spinal control of gait bypassing the impaired vestibular and/or cerebellar inputs (26). However, as Figure 2 suggests, this may be a purely mechanical phenomenon.

### Dynamics of the Path of the Center of Mass: The Three-Dimensional Curvature

**Figure 3:**
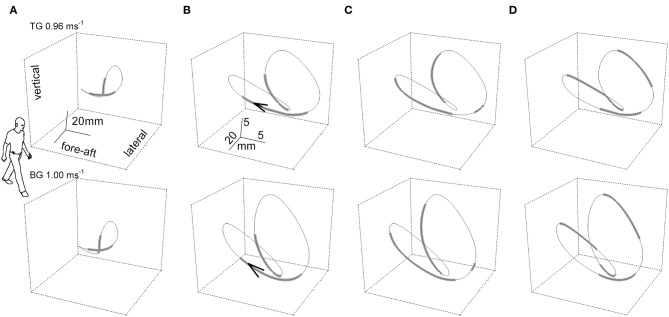
The “bow-tie” path of the body center of mass (CoM) averaged over a series of strides. In the upper and the lower row of panels, two representative subjects walked at 0.96 and 1 m s^−1^, respectively. Subject TG (upper row column) was a man, 23 years of age, 1.78 m in height, 74 kg in weight with a leg length (superior iliac spine to ground) of 0.91 m. Subject BG (lower row) was a man, 30 years of age, 1.73 m in height, 66 kg in weight with a leg length (superior iliac spine to ground) of 0.88 m. Six subsequent strides were recorded per subject. In the **(A)** column, the curve gives the CoM path and thickened segments correspond to the double foot-ground contacts. The human form outline (arbitrary size) facilitates recognition of the spatial orientation of the figure. In columns **(B–D)**, the scaling of the curves is modified for graphic clarity. **(B)** Foot-ground contact phases. The arrow tip marks the conventional beginning of the stride; i.e., the point where the forward speed reaches a maximum after the right foot strike. **(C)** Thickened tracts mark the segments of the trajectory where the curvature is classified as low, at or below 0.05 mm^−1^ (i.e., radius of curvature at or above 20 mm). **(D)** Thickened tracts mark the segments of the trajectory where the instantaneous recovery of mechanical energy, R_inst_, is at or below 0.7. It can be seen that the phases of high curvature (thinner tract in **C**) largely overlap the single stance phases (thinner tracts in **B**), and phases with low values of energy transfer (R, thicker tracts in **D**). Taken from Tesio et al. (27), used with permission.

The dynamics of the CoM path, that is, its trajectory, provide more information than the morphology. The instantaneous curvature, which is the inverse of the radius, and the tangential speed were measured along the bow-tie, in parallel with the instantaneous values of the recovery of mechanical energy (R_inst_) as a function of the distance (arc-length) covered during one stride (27). As Figure 3 shows, one phase of external power production falls within the push-off period around the double stance (see below for further description); while the other phase is approximately coincident with the inversion of the lateral oscillation in the middle of the single stance (Figure 3).

**Figure 4:**
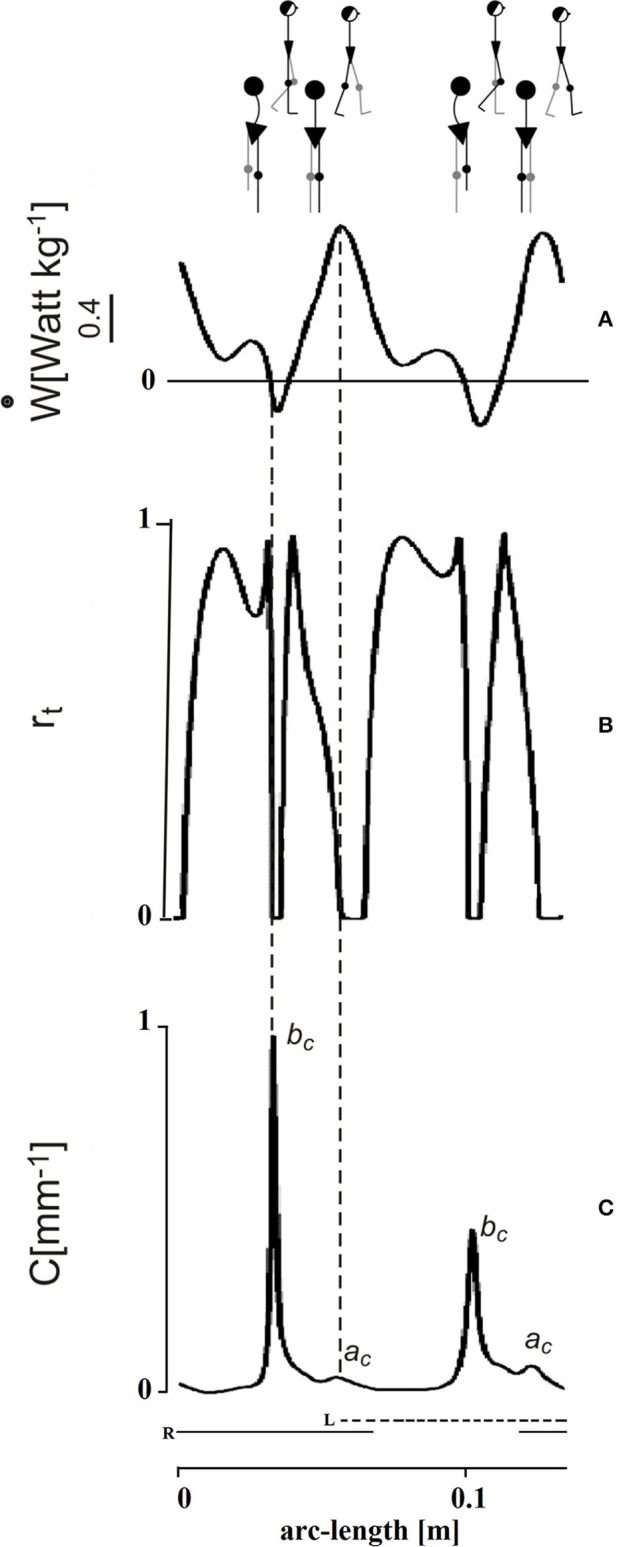
Mechanical phenomena along the bow-tie trajectory of the center of mass (CoM). The tracings depict one stride performed by one subject walking at 0.96 m s^−1^ (see the human sketch on top). The abscissa gives distance (arc length of the CoM path). The time needed to cover the whole represented arc-length distance was 1.15 s (not shown). Left (L) and right (R) foot stance is represented by the dashed and continuous horizontal bottom segments, respectively. Conventionally, the initial point of the tracing corresponds to the maximum forward speed of the CoM shortly after the right heel strikes the ground. **(A)** In the top panel, the ordinate gives the external power (ẇ) subtending the changes of mechanical energy of the CoM. This indicates active muscle shortening to increase the total mechanical energy of the CoM, while negative power indicates muscle contraction during elongation, to absorb the loss of total energy, assuming no frictional resistance. In either case, the pendulum-like mechanism is less than optimal. **(B)** Index of the pendulum efficiency. The recovery of total mechanical energy, r_t_ on the ordinate (R_inst_ in the text) gives the instantaneous changes of E_tot_, hence the instantaneous muscle work saved as a fraction of the summed changes of kinetic and potential energies (0 = no recovery; 1 = complete recovery). Zero values (no recovery) imply that the gravitational potential and kinetic energies of the CoM change in phase, no matter whether toward increase or decrease, so that no pendulum-like transfer is possible and muscle intervention (positive power during shortening or negative power during lengthening, respectively) is required. **(C)** Graph of the curvature of the trajectory. There are two impressive drops to zero of r_t_ during each step. These are coincident with peaks of curvature along the CoM trajectory. The smaller curvature peak (*a*_*c*_) occurs during the double stance and is roughly coincident with the a increment of E_tot_ (see Figure 3A), when the plantar flexors make a strong injection of energy into the body system. The larger peaks of curvature (*b*_*c*_) occur during late single stance and are strikingly coincident with the peak of a brief burst of negative external power, just before the push-off (production of positive power, see panel **A**). The *b*_*c*_ peaks highlight the braking of the CoM along the lateral direction, preceding its redirection. Adapted from Tesio et al. (27), used with permission.

The time course of R_inst_ during one stride is presented in Figure 4 (second tracing from top). This course appears jerky: R_inst_ remains above 70% for much of its path and peaking close to 100%, a value which indicates a fully ballistic pendulum-like motion. There are two short phases of the step, however, when R_inst_ drops suddenly to zero and the curvature (bottom curve) shows evident peaks. The larger peak occurs during the single-stance period (labeled *b*_*c*_ in Figure 4), around the lateral redirection of the CoM. Incidentally, these curvature peaks may differ between the left and right step, as in the present case. Smaller curvature peaks occur during double stance (*a*_*c*_ in Figure 4). Interestingly, while *a*_*c*_ is coincident with a peak of external power (uppermost curve) as would be expected), the larger *b*_*c*_ curvature peak is synchronous with a narrow peak of negative external power (i.e., power absorbed by muscles that are active during elongation). In other words, the lateral oscillation of the CoM during the single-stance period requires active braking, followed by active propulsion (no passive energy exchange, R_inst_ = 0). These events allow the CoM to be actively redirected toward the opposite side. It is known that lateral-externally oriented ground reaction forces occur throughout the entire double-stance and early single-stance periods but not during late stance (28). The internal redirection during single stance is thus a critical phase of the step, in which the passive-ballistic motion must be suddenly and briefly superseded via active neural control imposing a small and swift absorption of external power in order to brake the CoM, just before the active redirection toward the opposite side. If this mechanism fails, a lateral fall might ensue.

### Trajectory of the Center of Mass and Balance

The peaks of curvature of the CoM path and its lateral oscillations provide an interesting focus for the assessment of balance during walking. Balance deficits can result in falls mostly during walking, and mostly into the lateral direction (29). Lateral instability is, therefore, a likely marker of future falls. A relevant study demonstrated that in forward-induced stepping the main difference between elderly faller and young or old non-fallers is the greater sideways motion of the body toward the stepping side and a more laterally directed foot placement. Potential fallers thus seem to pre-plan this protective behavior (30). The hypothesis of careful neural control of lateral displacement of the body is in line with findings on treadmill walking, where healthy subjects adopt a wider base of support, compared to ground walking (see Note S3). This notwithstanding, the lateral margins of stability (the distance between the outer margins of the base and the vertical CoM projection, see below) are kept unchanged (31). Therefore, an understanding of the CoM trajectory and its lateral width during the stride has high clinical relevance. A more precise analysis of the risk for fall may be possible through knowledge of the spatial relationships between the position of the CoM and the base of support (both uni- or bi-pedal) in dynamic conditions. A seminal paper (32), defined the vector quantity, extrapolated CoM (XCoM), using Equation (1):

(1) $$\begin{array}{r} XCoM=CoM_{position}+CoM_{velocity}*\sqrt{\frac{l}{g}} \end{array}$$

where *l* is the lower limb length and *g* is gravitational acceleration. The margin of stability (MoS) is defined as the minimum difference between the perimeter of the base of support and XCoM. In adult walking, the lateral MoS is much lower than would be predicted by considering the CoM alone (say, 25 vs. 50 mm) (33). Computing the absolute position of XCoM with respect to the base of support requires knowledge of the base of support itself. The needed information can be available through kinematic analyses as will be detailed later in this review. Since the publication of Hof's paper in 2005, there have been plenty of studies investigating the size of the MoS, and even the “temporal” MoS, i.e., the time remaining before the MoS is trespassed, during walking in various populations of healthy subjects of all ages and under various paradigms, implying perturbations of the walking surface (30, 31, 34–40). From this literature, the general principles seem to emerge that lower margins of stability after perturbation indicate an actual risk for balance, while increased margins of stability indicate a protective attitude, reflecting a latent instability.

The potential clinical usefulness of the XCoM analysis, capturing the balance variable during walking, cannot be underestimated. Not surprisingly, technical efforts are ongoing to simplify its estimation during walking, e.g., through a limited set of optical markers (41) or even through wearable sensors placed under heel and toes (42).

### The Path of the Center of Mass in Children and Adolescents

A recent study aimed to relate the CoM path to age in children (5–13 years old) and adults (23–48 years old) using metric distances normalized to subjects' height. Walking velocity was also normalized to height through the Froude number (43). Both the bow-tie shape of the CoM path and its U-shape relationship with walking velocity were preserved across age groups, indicating that these parameters decrease relative to body elongation. Increased age and height were associated with shorter height-normalized paths, due to narrowing of the lateral dimension. This indicates that reduction of the lateral oscillations of the CoM relative to body height may be taken as an index of the neural maturation of gait, as already foreshadowed in a previous study (23).

## The Transport of the CoM as a Matter of Cost

### Mechanical Energy Changes in the Inverted Pendulum Motion

**Figure 5:**
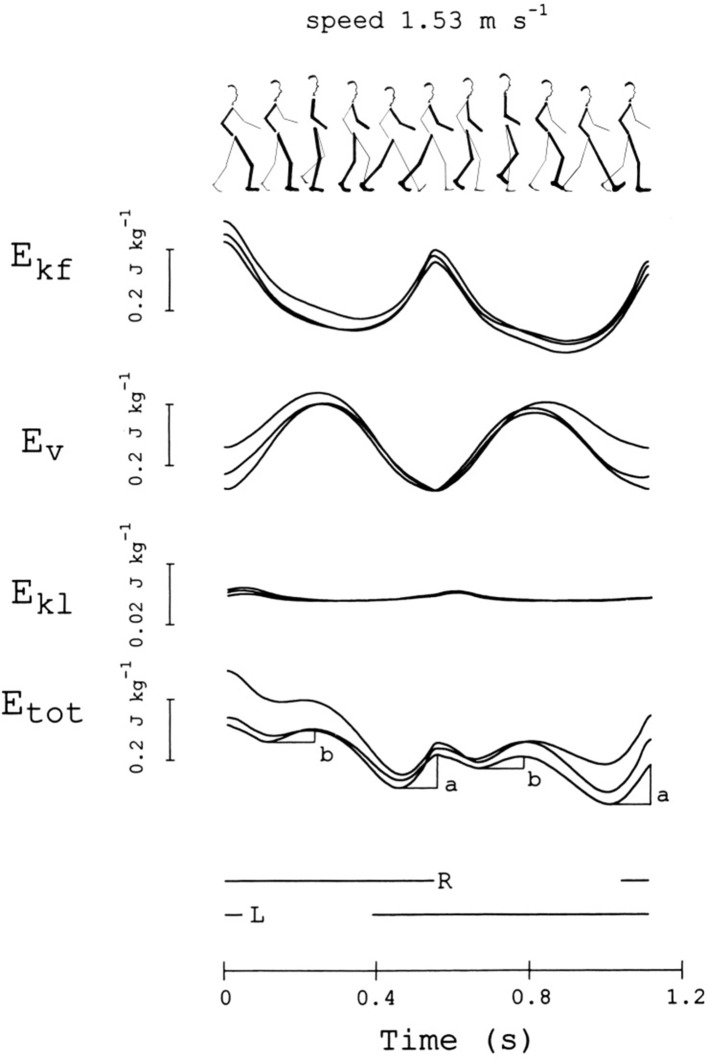
Changes in mechanical energy of the center of mass (CoM) during one stride. This subject (sketched on top) was a woman, 36 years of age, 1.5 m in height, 50 kg in weight, walking at 1.53 m s^−1^. From top to bottom, the curves refer to the mechanical energy changes of the CoM due to motion in the forward, vertical and lateral directions (E_kf_, E_v_, and E_kl_, respectively), and their sum, E_tot_ = E_kf_ + E_v_ + E_kl_ (note the scale difference for E_kl_). The stride is considered to begin when E_kf_ reaches a maximum. Curves from three different strides, performed at about the same average speed (±10%), are superimposed. The bottom horizontal lines mark the time intervals in which the right or the left foot is on ground. The a and b labels indicate the increments of E_tot_, necessarily sustained by positive muscle work (external work, W_ext_). Adapted from Tesio et al. (20), used with permission.

Figure 5 gives a modern representation of the mechanical energy changes of the CoM in a healthy adult during a series of two subsequent steps (strides), beginning with the heel strike of the right, leading lower limb (ground contacts are marked by the interrupted horizontal segments below the curves). Data were obtained through the “double integration” method [(20), see Note S2].

The pendulum-like mechanism subtending the motion of the CoM is revealed from the time-course of the mechanical energy during a step. The mechanism allows the kinetic energy of the CoM, due to the forward velocity (E_kf_), to be transformed into the sum (E_v_) of gravitational potential energy (E_p_) and kinetic energy due to the upward velocity (E_kv_). The changes of kinetic energy due to the lateral motion (E_kl_) are much smaller than those observed in the sagittal and vertical directions. In an ideal pendulum the friction forces of the air and at the hinge are neglected, the time course of E_kf_ is the mirror image of the time course of E_v_, and the sum of these energies, E_tot_, remains constant; the oscillation would be perpetual and no work would be necessary to keep it in motion with respect to the ground (“external” work, W_ext_) (44). Of course, the mechanism is not ideal in walking, modeled as an inverted pendulum. Figure 2 shows that the energy exchange is not complete, although oscillations of E_tot_ are very limited. Actually, the mechanism allows a remarkable saving (Recovery, R) of W_ext_. R can peak at 100% (no muscle work required) and reach 60% as a mean along a step, at “optimal” velocities around 1.3 m s^−1^. Increments of E_tot_, necessarily sustained by muscles, occur around the double stance (increment a) and during the single stance (increment b) (fourth curve from the top in Figure 5). These concepts are recalled in the Note S4, and further on in the main text.

Minimizing the cost of transport of the CoM seems a primary constraint of the walking mechanism. The average velocity selected spontaneously and, for any velocity, the step cadence and length, are very close to those allowing to minimize the total energy expenditure and W_ext_, and to maximize R (see Note S4 for details). Also, the inverted pendulum mechanism is tenaciously maintained across all ages (see following paragraphs).

### The Inverted Pendulum During Walking in Children

Children aged 1–4 years are rarely capable of maintaining a constant average velocity during walking. This makes the estimation of velocity-dependent energy changes of the CoM very difficult. In general, it is agreed that a pendulum-like motion of the CoM appears around the age of 2 years. Estimates with large margins of uncertainty suggest that this mechanism achieves adult-like characteristics very soon thereafter (45). Above 4 years of age, mass-specific metabolism and weight-adjusted mechanical external power are higher for children than for adults when compared at the same walking velocity (46). However, this difference vanishes when velocities are compared after adjustment for the subject's height through the non-dimensional Froude number (46, 47). This suggests that the assumption of child-adult geometric similarity, justifying the use of the Froude number, is tenable despite some concerns raised in the Literature (48, 49).

### The Inverted Pendulum During Walking in the Elderly

The effects of aging in subjects 65 years or older on various kinematic and dynamic walking parameters have been studied extensively. In two recent and robust meta-analyses, the impact of aging appeared modest (50, 51). In general, elderly people tend to spontaneously adopt a lower speed and a lower step length for any given speed compared with younger adults. This means that disentangling the effects of aging from those of forward velocity and step length with respect to dynamic parameters is challenging (52). In general, lower propulsion from the trailing leg, a lowering of the forward CoM accelerations, a decrease in braking the fall of the CoM at foot strike and a restriction of the mediolateral CoM sway seem to characterize the CoM behavior in individuals above 65 years of age (53, 54). The increase in forward speed precedes that of the upward velocity, as happens at lower speeds. The graph of vertical to forward velocity, the “hodograph” (28), was proposed as a potential index of age-related decline in gait performance. It should be noted, however, that in reality, elderly subjects compensate their limits by adopting a shorter step length (see the above considerations on this point). A study of dynamic balance during walking demonstrated that the mediolateral displacement of the CoM was comparable in elderly and young subjects (55), with a difference that elderly subjects exhibited a cautious narrowing of this displacement when the base of support was restricted. Also, the variability in CoM mediolateral velocity was found to be higher in elderly subjects. The search for a safer motion of the CoM has been suggested as the reason that elderly people, but not younger ones, decrease their average forward velocity, stride length, and minimum forward CoM velocity during the single-stance period when wearing socks compared with walking barefoot. This cautious behavior seems to counteract the known age-related decrease in plantar foot sensitivity (56).

## Matching the Systemic and Segmental Views of Walking. Muscles and the Translation of the CoM

Given that minimizing the cost of transport is a primary constraint of the CoM motion, the neural control of gait must be designed accordingly.

### When Is W_ext_ Produced?

In recent years, a small number of studies have measured the instantaneous changes of E_tot_ (i.e., W_ext_) and R_inst_ (27, 57). These studies paved the way to the understanding of the role of individual muscles in generating the motion of the CoM. This information first requires knowledge of the timing of W_ext_ output.

An early seminal study (44) demonstrated that W_ext_ is provided in two reproducible phases of the CoM oscillations along the sagittal plane; specifically, when increases in E_kf_ (named increment a) occur and when the CoM is lifted (increment b). Both phase shift and size differences between its components may lead to increments of E_tot_. In general increment a is due to the fact that E_kf_ increases more than Ev decreases, while the opposite is true for increment b (44). A successive study demonstrated that the peak external power (work/time ratio) during increment a is about four times higher than that required to sustain increment b (20).

### Matching Segmental and CoM Power Production

Unfortunately, reports of the synchronous recording of motions of body segments and the CoM are rare. One practical reason is that parallel independent platforms are needed to record joint moments and torque during left and right leg stance, and platforms which allow at least one entire stride are required for the double-integration recording of CoM motion. In addition, a conventional segmental gait analysis must be also available simultaneously. In general, research questions were mostly focused on segmental or CoM motion rather than on their interaction, and some key questions have only started to be answered recently. The segmental approach conventionally defines step periods as the time interval between successive foot-ground contacts, whereas Cavagna's studies considered a step to be the interval between successive peaks of E_kf_, thus presenting a fully CoM-based concept of “cycle.” This makes a comparison of segmental and systemic events between studies difficult.

**Figure 6:**
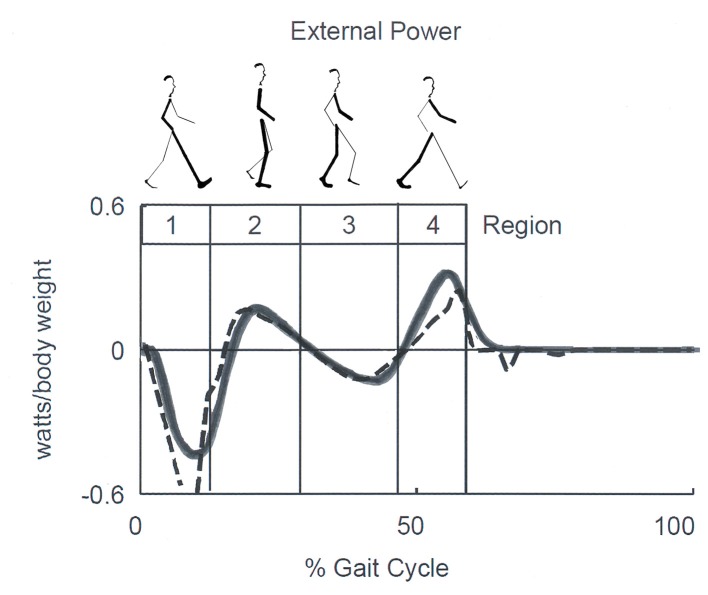
Sketch of Neptune's Regions of the step. Regions are defined by the zero crossing of the external power curve, which corresponds approximately to double support in Regions 1 and 4, and to the rise and fall of the center of mass (CoM) during single stance in Regions 2 and 3, respectively. Late Regions 3 and 4 correspond to the push-off phase according to other authors. Continuous and dashed lines correspond to experimental and model-simulated data. Adapted from Neptune et al. (58), used with permission.

In recent years, a correlation between the E_tot_ changes and temporal step phases was formally proposed. Neptune et al. (58) identified four temporal phases dubbed “Regions.” These were based on the zero-crossing of the external power applied to the CoM during one step: Region 1, the step-to-step transition of the leading leg as it absorbs the mechanical energy of impact (approximately the first phase of double support); Region 2, the raising of the CoM in early single-limb support; Region 3, the lowering of the CoM in late single-limb support, and Region 4, the step-to-step transition of the trailing leg as it propels the body (approximately the second region of double support) (Figure 6).

By comparing Figures 5, 6, it can be seen that Cavagna's increments a and b (see also Figure 2) occur approximately during the step-to-step transition of the trailing leg (Region 4) and the early single-limb support (Region 2) phases, respectively. Hereinafter, the labels “increment a” and “b” will be adopted preferentially, although they will be also referred to as the Neptune's stride phases of their occurrence. In line with the literature (59), the term “push off phase” will also be used as a proxy of “step-to-step transition of the trailing leg.”

### The Dominant Role of Plantar Flexors in Generating Wext

**Figure 7:**
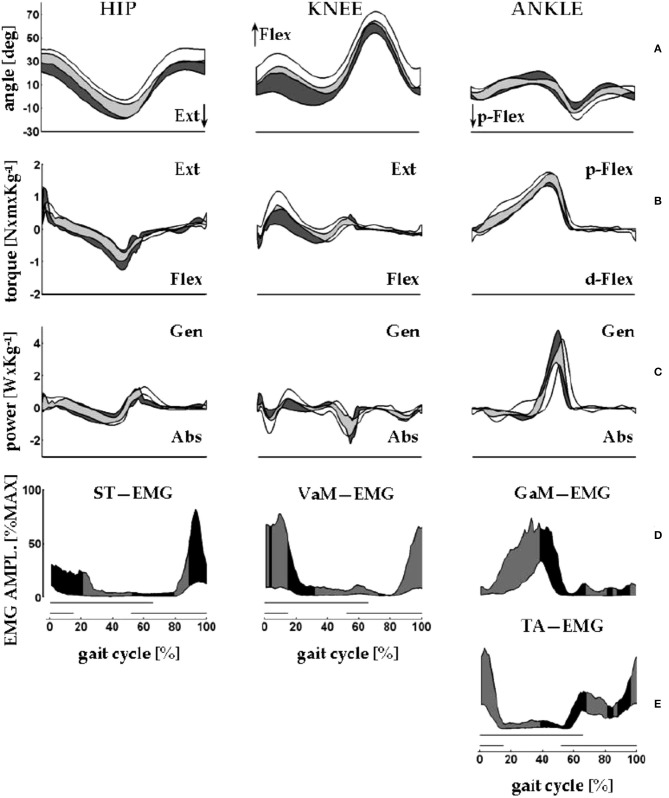
An overview of measurements of lower limb motions from eight healthy adults (four men) during walking on a force-sensing treadmill. Average velocity: 1.32 m s^−1^; average cadence: 125 steps min^−1^. The x-axis gives the standardized stride time (data interpolated in 100% points; average absolute stride duration = 0.96 s). Data from 10 strides were averaged. Here, the beginning and the end of the stride are made to coincide with the right foot strike. Dashes on the bottom of each column of panels mark the average duration of the stance phase of the stride under examination (thick upper line) and of the simultaneous contralateral stride (thin lower line). **(A)** From left to right, the y-axis shows sagittal rotations of hip, knee, and ankle joints. **(B,C)** From left to right, the y-axis shows the joint torques and powers, respectively, of the hip, knee, and ankle joints. Joint power is labeled as generated (Gen) or absorbed (Abs) when the velocity of joint rotation and the joint torque have the same or opposite directions, respectively. During swing, torque and power at the lower limb joints are estimated through inverse dynamics according to anthropometric modeling of body segments. In each panel, the black band encases the mean ± standard deviation of the control sample (walking over ground), whereas the white bands encase the mean ± standard deviation of the sample (walking on the treadmill). Gray bands indicate the overlap between the data from the study (white and gray areas) and control samples (black and gray areas). **(D,E)** The electromyographic (EMG) signal envelopes from the semitendinosus (ST), vastus medialis (VaM), tibialis anterior (TA), and gastrocnemius medialis (GaM) of the right lower limb. For each muscle, amplitudes were standardized as percentages of the maximum voltage recorded from that muscle across all of the recorded strides (in microvolts: GaM, 174; TA, 197; VaM, 64; and ST, 114). Each band encases the 10th to the 90th percentiles of the EMG amplitudes from the corresponding muscle. The black and gray fillings closely follow the shortening and lengthening periods of the corresponding muscle, respectively. For ST, VaM, TA, and GaM shortening is assumed when the hip and knee are extending and when the ankle is dorsally or plantarly flexing, respectively. Sampling frequency for kinematic and dynamic data: 250 Hz. Treatment of the EMG signal: pre-amplification, 16-bit A–D conversion; sampling frequency: 1 kHz, rectification; smoothing through moving average: 80 ms window. Taken from Tesio and Rota (65), used with permission.

Clinical applications of knowledge of the CoM motion requires inferences on the underlying role of segmental impairments. In this view, the synchronous observation of changes of E_tot_ (i.e., W_ext_, which is positive when E_tot_ increases) and of muscle recruitment appears highly relevant. The sequence of activation of lower-limb muscles can be recorded using surface electromyography or fine-wire implanted electrodes (60). This sequence is well-known for adults (61, 62) and children (63) and is reported in many manuals on gait analysis [such as Figures 8.17, 8.18 of (64)]. These studies provided evidence that the plantar flexors are active, both mechanically and electrically, from midstance to mid push-off, Neptune's Regions 3 and 4, respectively (Figure 6), while the ankle dorsal flexors are active during early stance and late push-off, Regions 1, 2, and 4, respectively. Only minor activity is recorded from the knee and hip extensors, mainly at foot strike (Figure 7) (65).

**Figure 8:**
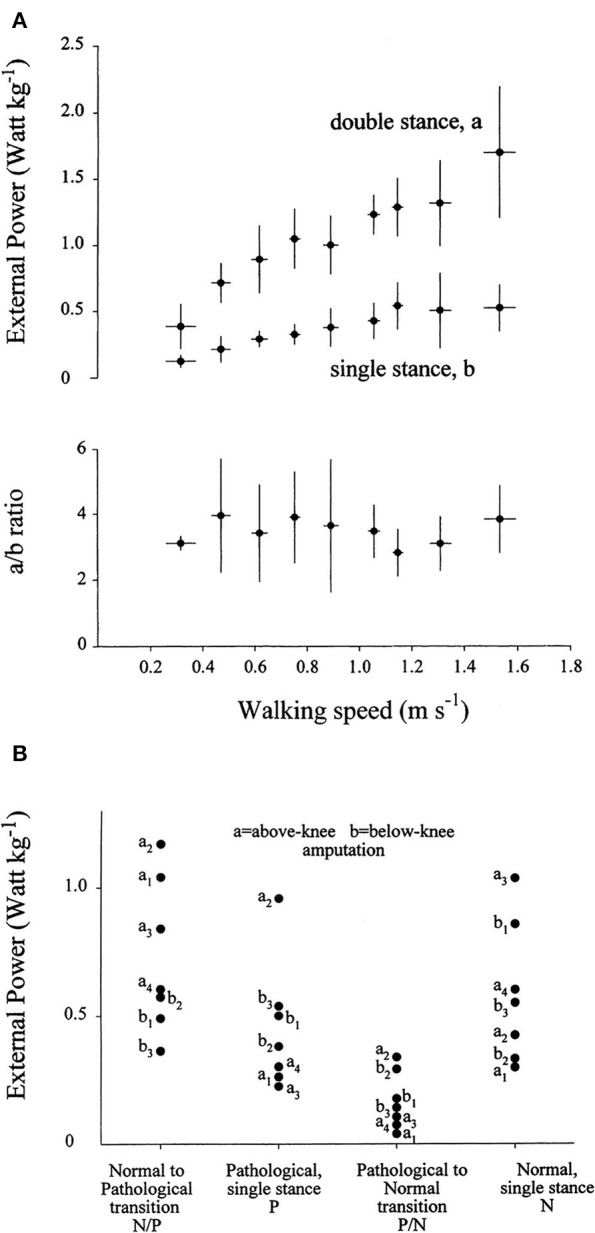
**(A)** The upper panel gives the average of the weight-normalized external power provided by muscles to sustain the increments of the total mechanical energy of the center of mass (CoM) (E_tot_, see Figure 5) during walking at different speeds (on the abscissa). Power was measured during the increments of E_tot_ that occur during the double- and single-stance phases of the step (a and b, see Figure 5). Lower panel: the a/b ratio plotted as a function of speed. Power is invariably higher during the double-compared with the single-stance phase of the step. Taken from Tesio et al. (20), used with permission. **(B)** The ordinate gives the average power provided by muscles to sustain the a and the b increments (during double and single stance, respectively) of the total mechanical energy of the CoM (E_tot_, see also Note S5) during one entire stride in three below-knee (dots labeled b1–b3) and four above-knee (dots labeled a1–a4) amputees. The N and P labels indicate the single-stance phases of the stride on the normal and prosthetized lower limbs, respectively. The N/P and P/N labels indicate the double-stance phases of the stride (normal limb behind or prosthetic limb behind, respectively). During the N to P transition, much more power is generated when the unaffected limb is trailing, compared to when it is leading. Taken from Tesio et al. (66), used with permission.

The mechanical role of plantar flexors seems crucial, given that they are synchronous with the a increment of E_tot_, which is 3 to 4 times higher than the b increment at all velocities (Figure 8A) (20). In the asymmetric impairment caused by above- or below-knee amputation, the a increment is increased when the unaffected limb is trailing (N/P transition in Figure 8B); correspondingly, the plantar flexors may generate a peak power 3 times higher, compared to the next a increment sustained by the muscles of the amputated limb (P/N transition in Figure 8B) (66).

**Figure 9:**
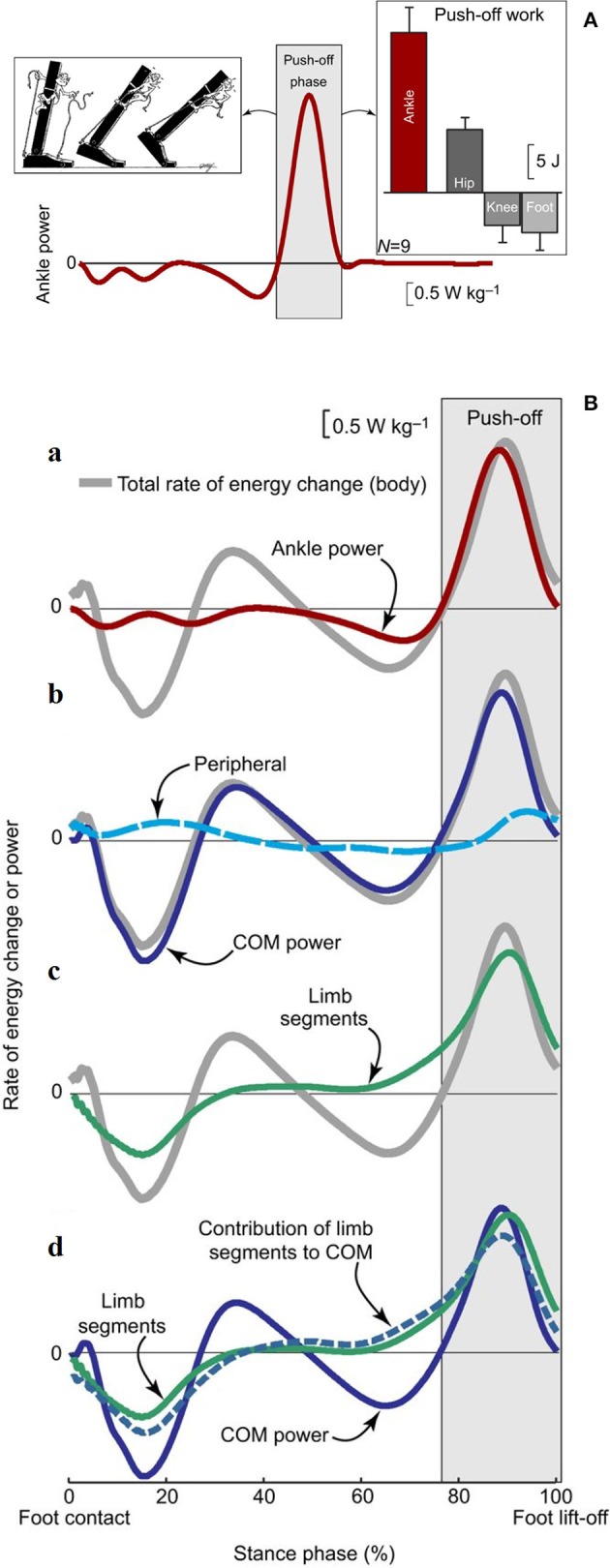
**(A)** Ankle power for one leg plotted across a full stride cycle, from foot contact to subsequent ipsilateral foot contact. The left inset depicts the ankle push-off behavior. The right inset, ankle push-off work vs. the push-off work performed by other ipsilateral lower-limb joints and segments (hip, knee, foot). Work values were obtained by computing the time integral of power during the push-off phase. Mean inter-subject power and work values and work standard deviations are depicted for walking at 1.4 m s^−1^ based on 6-degrees-of-freedom joint mechanics analysis (*n* = 9). Taken from Zelik and Adamczyk (59), used with permission. **(B)** Rate of energy change or power (work rate) estimates for an individual limb during human walking. The integrated area under each curve during push-off (light gray box) represents the magnitude of push-off work or energy change. (a) Ankle power (red line) overlaid on Total rate of energy change (gray line, due to the motion of and about the body's center of mass [CoM]). (b) The majority of total rate of energy change during push-off is attributable to CoM power (blue line, defined here as the rate of energy change due to push-off limb power production), and smaller contributions are from peripheral energy changes (due to segmental motion relative to the CoM; dashed cyan line). (c) The majority of total energy changes during push-off is also attributable to segmental changes from the push-off limb (green line). (d) The contribution of limb segmental energy changes (green line) to overall CoM changes (solid blue line) is shown here as a dashed blue line. During push-off, the majority of the limb power goes into this contribution, which in turn accounts for the majority of CoM power. Data depicted are inter-subject means at 1.4 m s^−1^ (*n* = 9). Taken from Zelik and Adamczyk (59), used with permission.

Recent research confirmed that the plantar flexors provide the highest power output during walking compared with other extensor muscles that act at the knee and hip (64, 67). This power is mostly released during the push-off phase and is thus expected to have a critical role in the advancement of the body system (68, 69). Active stretching of the plantar flexors occurs midstance, immediately before the push. This has led some authors (70, 71) to suggest that the plantar flexors group or at least the soleus (72) are primarily responsible for a braking action with respect to the CoM fall in late stance (Figure 9A).

Other authors proposed that the plantar flexors are used to accelerate the leg into the swing (73, 74), thus producing W_int_. Given the methods that were used, these segmental interpretations did not consider the systemic role of the plantar flexors; however, they are compatible with a systemic interpretation. For instance, the braking action of the stretched plantar flexors during active lengthening cannot be ignored, yet it can be interpreted simultaneously with a mechanism that facilitates the remarkable production of positive ankle power during the successive shortening (69). Consistently enough, the work produced by these muscles has been shown to be four times more efficient than the work produced by the hip muscles to sustain the b increment of E_tot_ during the single-stance period, the pendular phase of the step (68). This highlights the contribution of the braking action of calf muscles to the next increment a of E_tot_ during Region 4 of double support. Meanwhile, their role in raising the mass of the swinging leg contributes to the lift of the CoM (59), and thus, to increment b of E_tot_, during the subsequent early single-limb support (Region 2). These studies show that appropriate modeling of positive work (i.e., when force is produced by shortening muscles) at lower-limb joints, considering both their rotational and translational energies, can explain the increments of E_tot_ along a stride (75) (Figure 9B). In short, both the segmental and system viewpoints highlight the role of the push-off as the critical phase when muscle power is generated within the walking body system. There is converging evidence that the plantar flexors are the major “engines” involved in sustaining the increment a of E_tot_. Their contribution to leg swing means that they also contribute (together with knee and hip extensors) to increment b. This is confirmed by the increased power production during ankle plantar flexion compared with other sagittal joint rotations (67) and the increased power production during increment a compared with that of increment b of E_tot_. Clinical observations confirm this conclusion, as is discussed in detail later in this review.

### The Role of Lower Limb Muscles in Children

The pendulum-like transfer of the CoM has adult-like characteristics from the age of 4 years, once the body height is conditioned out by transforming the average walking velocity into the non-dimensional Froude number. The only one exception is perhaps the wider lateral size (relative to body height) of the 3D path (the “bow-tie”) of the CoM. Timing of joint power and electromyographic patterns of the lower limb muscles also seem independent of age (76).

### The Role of Lower Limb Muscles in the Elderly

Elderly people (say, above 65 years of age) tend to adopt a lower average velocity and, for any given velocity, a shorter step length, compared to their adult counterpart. These features appear to compensate for weakness, in particular, of the plantar flexors, and for subclinical balance impairment. A recent study confirmed the existence of age-related differences in terms of propulsion from the trailing leg (77) by comparing young subjects (mean age 24 years) with a sample of elderly subjects (mean age 74 years). The power of the plantar flexors was reduced in the elderly group at the step-to-step transition, which indicated that at speeds above 1 m s^−1^ elderly subjects were unable to simultaneously increase the velocity of the CoM forward and upward. Once the lower velocity and lower step length were taken into account, the lower-limb joint dynamics and estimated roles of individual muscles in propulsion of the CoM were found to be superimposable between young (24 years old on average) and elderly (74 years old on average) subjects (52). More recently, however, evidence has been reported for a distal-to-proximal redistribution of positive work across the plantar flexors and knee and hip extensors in elderly people (mean age 76 years) compared with younger people (mean age 22.5 years), when tested at the same walking velocities, during both level and uphill walking (78). The proximal shift of work production was higher with higher velocities. This is consistent with the fact that plantar flexors, compared with knee and hip extensors, work at closer to their maximum capacity (79). As a consequence, general age-related strength loss (80) may prevent the plantar flexors from providing the amount of work that they release during walking at a younger age. This notwithstanding, plantar flexors of elderly individuals never provide <50% of the positive work done by the lower-limb muscles during level walking, and thus they remain the principal engines of walking at any age (78).

In summary, once the inverted pendulum mechanism is established, the role of individual muscles in CoM motion seems to remain largely unchanged from infancy to old age although the plantar flexors seem to become less dominant compared to the proximal muscles at advanced age. The main adaptations of walking to body size and shape, muscle strength and joint mobility are related to velocity and the step length that is adopted and attainable (50).

## Clinical Significance of the Motion of the Center of Mass During Walking

Human walking impairments are varied but, for simplicity, here they can be divided into symmetric and asymmetric forms, depending on the mechanical events observed during each of the two steps within a stride. A key concept, nearly a paradox, emerging from the Literature is that walking impairments may be associated with an overall normal total energy expenditure per unit distance, which in turn reflects a normal efficiency of the pendulum-like mechanism as an average between subsequent steps. Evidence is provided in the following paragraphs and is further discussed in Note S5.

### Symmetric Pathological Gaits

Most symmetric diseases and impairments, including many forms of Parkinson's disease, infant cerebral palsy, incomplete spinal lesions, and multiple sclerosis, typically involve a lower velocity during walking. Given the velocity adopted, a normal W_ext,m_ and/or a normal 3D path of the CoM (see below) can be observed in symmetric impairments. This has been found to be the case in scoliosis (81), multiple sclerosis (82), obesity (83), non-freezing Parkinson's disease (84), and hemophilic arthropathies (85). In most pathologic conditions, steps tend to be even shorter (hence, their cadence higher) than is physiologically necessary for the lower speed that is adopted. In extreme cases, the feet never leave the ground in a so-called “shuffling gait” (86). Shortening the steps raises W_int,step_ (87) at all speeds, yet it minimizes W_ext, step_. In fact, smaller fluctuations in E_tot_ are seen. This means that the power required from the plantar flexors at each step is decreased, as is the need for balance control given that accelerations of the CoM are minimized (see also Note S4). For these reasons, shortened, and thus more frequent, steps flag (non-specifically) an underlying difficulty negotiating the advancement of the body system. A very simple, yet reliable clinical index is provided by the ratio of step length to step frequency, which is referred to as the walk ratio, where a higher value indicates greater control (88–90).

### Asymmetric Pathological Gaits

Knowledge of CoM motion is particularly useful for the clinical assessment of asymmetric gaits. Various terms are used to describe gait asymmetries, which are based on a visual inspection and generally defined as limping or claudication. In clinical jargon, “escape” limp indicates that the single-stance period is minimized for the impaired lower limb with the opposite limb being brought to contact the ground quickly to unload the impaired limb in anticipation of its take-off. This allows the patient to keep the impaired limb off the ground for as long as possible. This is perhaps the most sensitive clinical sign of asymmetric impairment (see Note S6) (91), although it is non-specific because pain, limited joint mobility, ataxia, weakness, or spasticity may all result in an escape limp. Furthermore, step length asymmetry may coexist. As a rule, the heel-to-heel sagittal distance during the double-stance period is longer when the affected limb is leading, also known as the anterior step, compared with when the unaffected limb is leading. This provides a visual index of the reduced propulsion from the impaired lower limb as its role in CoM propulsion is fostered by the rear position. Other clinical definitions refer to the most significant segmental alterations: “sickling” gait refers to exaggerated abduction of the swinging leg, usually due to reduced knee flexion (sometimes, however, due to foot drop); “steppage” refers to foot drop during swing, which leads to excessive hip flexion (but sometimes to sickling), and the old Trendelenburg's sign (91) refers to the sideways downward tilt of the pelvis on the swinging side, usually due to weakness of the hip abductor muscles of the supporting limb. All of these signs may coexist in the case of an escape limp. There may be cases, however, in which neither visual inspection nor the instrumental recording of kinematic variables provide sufficient, or any, information despite the presence of large dynamic asymmetries at the levels of the joints and the CoM. The patients presented in the figures above, suffering from hemiparesis, hip arthritis, above- and below-knee amputation and knee rotationplasty, respectively, all walked with a nearly normal appearance. Nevertheless, the motion of the CoM was very asymmetric; the body system “pole-vaulted” almost passively on the affected limb, whereas extra work was required from the muscles during the “push” from the unaffected limb.

This agreement between segmental and systemic dynamic asymmetries is expected. However, from analyzing the results in the literature, at least three unexpected findings could be identified. The first is that the effects of most asymmetric diseases on the mechanics of the CoM may be surprisingly similar. The second is that when kinematic effects are minor, the asymmetries of the CoM motion may be large nonetheless. For instance, both in post-stroke (92, 93) and multiple sclerosis patients (94) a weak, if not absent, recruitment of the plantar flexors at push-off is observed. This allows sparing of the affected, or, more affected, lower limb. Given the force of the plantar flexors, their work output can be substantially abated with only a minor reduction of ankle rotation. The visual aspect of gait can be minimally affected while a strong asymmetry in W_ext_ and R between the subsequent steps is generated. As a third unexpected finding, there is a tendency for external work and power asymmetries to compensate between the two subsequent steps, with the result that means along the whole stride and hence, per unit distance, can remain normal. This raises a critical challenge to rehabilitation aiming at restoring symmetry, as will be clarified later on.

### The CoM Motion in Impaired Children

In adolescents with unilateral cerebral palsy, W_ext,m_ is only mildly increased (95). As for adults, symmetric impairments in children does not result in sharp differences in CoM motion compared with controls (96). The pendulum-like mechanism of translation of the CoM is less efficient (R_step_ is about 30% lower) in children with cerebral palsy compared with their healthy counterparts. However, it must be noted that patients with cerebral palsy walked more slowly and with a higher step frequency, making it difficult to assess the isolated effect of spasticity. A normal W_ext, step_ is obtained through a higher R_step_ in adolescents with mild obesity and Prader-Willi syndrome, as demonstrated by comparison of the Froude-normalized walking velocities of patients and controls (97). A paradoxical decrease of W_ext,m_ has been identified in adolescents who were operated on for scoliosis (81), who were also found to walk with a higher step frequency, hence, a shorter step length. This factor is, in itself, a source of decreased mechanical efficiency (87).

## The Enigma of “Willed” Asymmetries in Central Paresis. Implications for Rehabilitation Medicine

The affected lower limb of hemiparetic patients produces less work than it could potentially deliver. The unaffected lower limb is overloaded, and the CoM transfer motion retains a normal cost per unit distance. Given this successful adaptation, which rehabilitation approach might ever be effective?

### A Hidden Power Can Be Revealed in Various Walking Paradigms

Asymmetric patients can increase muscle work output from the impaired lower limb in at least four different conditions: at increased speeds (98), during uphill walking (99), during split-belt walking when the affected lower limb is forced to move faster than the unaffected lower limb (see below) (100), and by using crouch gait, also called bent hip-bent knee gait (67). In the latter, the increase in work involved the hip extensors more than the plantar flexors, while in the other conditions the greatest increase was invariably observed for the plantar flexors. More puzzling still is the fact that, whichever the velocity, the unaffected side also produced more work, thus maintaining the asymmetry. Asymmetry thus seems a goal rather than a necessity. This has been interpreted as a form of learned non-use (67), similar to that described for upper limbs after de-afferentation (101, 102) or stroke (103). Actually, this behavior looks automatic and unconscious, so that the adjective “acquired” seems preferable to “learned” (Baldissera, personal communication). A suggested treatment for upper-limb paresis is the forced use of the affected hand, which is induced by applying a constraint (e.g., a muffle glove) to the unaffected arm, which is termed constraint-induced movement therapy-CIMT (104). This approach is quite generalizable; its application to upper limb non-use was first described on monkeys in 1894 (101), and the analogous practice of eye patching to prevent amblyopia in strabismus is at least 2 centuries old (105). In the case of upper limb paresis, various forms of CIMT have been tested, associated with concurrent exercises and/or transcranial direct current stimulation (106). Despite the long-established research on upper limbs, the hypothesis of learned non-use of the lower limbs, and of tailored “forced-use” exercises, have only been proposed <2 decades ago (107). It is possible that the phenomenon has been overlooked for such a long time because unilateral impairments of the paretic lower limb are compatible with wide oscillations of this limb while walking. In the case of spasticity, the impaired limb may even appear overactive. Despite a visible swing, however, the affected lower limb always presents with a deficit in power production at the trailing ankle during late double stance, which lasts for about 10% of the step period (i.e., some 40 ms). The short duration and modest ankle rotations involved make it difficult to detect this dynamic impairment by visual inspection (however, see Note S6), although the recent introduction of force platforms has greatly enhanced detection.

In the authors' opinion, the learned/acquired non-use phenomenon is unspecific across asymmetric gait impairment, and independent of the underlying disease. This is indicated by the adaptation in both orthopedic and neurologic diseases, in which a normal value of W_ext,m_ can be achieved. This may explain the clinical observation that lower voluntary activation is associated with hypotrophy of the affected lower limb in hemiparesis (108), and with the invariable hypotrophy after amputation, including stump and thigh hypotrophy after trans-tibial amputation (109), contrary to the hypertrophy that would be expected from compensatory overuse. In support of this hypothesis, there has been at least one report on knee rotationplasty where hypotrophy was accompanied by a decrease in the cortical motor map area on the contralesional hemisphere, while an increase of this area was observed in the homolesional hemisphere (110).

### Restoring Symmetry Through Exercise: A Conceptual Challenge for Rehabilitation in Walking Impairments

Is forced-use of the impaired lower limb both a feasible and useful therapeutic strategy in asymmetric gaits? Both in stroke and lower limb amputee patients the impaired limb retains the capacity to provide the power sufficient to achieve a symmetric gait (67, 69, 111): nevertheless, power asymmetry is preferred. By sparing the affected side, asymmetry may minimize local consequences of limited joint mobility, paresis, and sensory deficits causing weakness, pain, and/or instability. Perhaps unfortunately, this adaptation is also compatible with efficiency in terms of W_ext_ (112) and total energy cost per unit distance (111). There seems to be no energetic motivation to restore symmetry. Power asymmetry, however, does have a clinical cost in the long run, making recovery of symmetry a desirable goal. Such clinical cost may occur in terms of impaired postural control at the “spared” lower limb, as demonstrated for standing (113) and gait initiation (114). Orthopedic troubles usually follow: a relevant example is provided by the frequent knee hyperextension (*genu recurvatum*) syndrome seen in various unilateral impairments, spanning from hemiparesis to cerebellar ataxia, to poliomyelitis, peripheral neuropathies, and myopathies. (115). Why are these long-term sequelae neglected? A pseudo-explanation is: because power (hence, kinematic) asymmetry can be achieved easily and sooner, thus fostering survival and therefore reproduction in a wild environment (105). Asymmetric walking may become soon compatible with normal velocity and cost of transport of the body system. Teleological explanations, including those resting on the Darwinian model of evolution, are not testable experimentally and therefore are pure conjectures yet, they are still part of the process of generating hypotheses (116). Whatever the reasons, reverting the tendency to retain asymmetry seems an appropriate goal, yet a very difficult challenge, for rehabilitation medicine. Original forms of exercise are needed, making at great disadvantage the asymmetric gait, perhaps under both the energetic and the balance perspectives of the CoM motion, compared to symmetric gait. Various approaches have been proposed based on orthotic hindrances (107), lower limb perturbations (117), and split-belt walking (118). The latter approach will be dealt with in the following paragraphs.

## An Emerging Idea: the Split-belt Treadmill as a Symmetrizing Tool

### Acquired Non-use of the Impaired Side May Affect Walking

Sparing (or “non-use”) of the impaired lower limb in the case of asymmetric walking has a deeply studied precedent in strabismus. A child that squints will commit visual acuity to the unaffected eye. Amblyopia will be much more likely than realignment. On the other hand, monocular vision will be more effective, from a behavioral standpoint, than diplopia. The analogy with the non-use observed in case of upper or lower limb impairments is quite strong (105). Maintaining vision of the squinting eye is based on the well-established approach of patching the unaffected eye, adopting a constraint-induced/forced-use rehabilitation paradigm (104). Split-belt walking represents a therapeutic analogy, which thus deserves discussion.

### Forcing the Use of the Impaired Lower Limb Through Split-Belt Walking

In split-belt walking, the subject walks on a force treadmill constructed with two parallel independent belts that can rotate at different velocities, hence the terms split- or dual-belt treadmill. Velocity ratios between the belts range from 1.5 to 4, depending on the study. Splitting causes a sudden escape limp in healthy subjects; within a few strides, the stance time and the “anterior” step length are reduced on the faster belt. The split-belt paradigm was introduced in a study on the capacities for early adaptation of the (supposed) spinal circuitries which underlie human walking (119). More recently, the cerebellum has been suggested to have a key role in consolidation of the treatment effect, specifically the after-effect (see below) (120). The idea of using this walking modality as a treatment for hemiparesis and Parkinson's disease has been proposed [for a review, see Reisman et al. (121)]. Step length was the main focus of most studies. The asymmetry in belt velocity may force symmetry between the length of subsequent steps, if the impaired (or the more impaired) limb—which usually shows a longer anterior step—is placed on the faster belt. When belts return to an equal velocity, or ground walking is commenced, an after-effect develops: the original asymmetry is attenuated or even reversed. For this reason, in agreement with the error-augmentation pedagogic principle (122), most researchers consider the “paretic slow/anterior step longer” configuration to be more promising (123).

### A False Analogy: Split-Belt Walking in Healthy Subjects Is Not Equivalent to Real Pathologic Claudication

The literature on split-belt walking as a rehabilitation treatment is increasing rapidly, despite the fact that some basic issues remain controversial. Initial studies were based only on kinematic observations. The analogy between experimental claudication in healthy subjects and true pathological claudication during ground walking is only valid for the stance duration, which is shorter for the faster leg of healthy subjects on the treadmill and for the paretic leg of patients during ground walking. This analogy is not valid for step length (see Video S1). Differences are even more relevant with respect to dynamic asymmetries between the lower limbs. The “escaping” leg, when placed on the faster belt, is required to produce extra power to avoid being dragged backward by the faster belt. In contrast, during ground walking, the paretic “escaping” lower limb provides a much lower power than the unimpaired lower limb (69). The technicalities and questions concerning the therapeutic use of this walking paradigm are discussed in Note S7.

Additional insight may come by raising the question: does increased symmetry of the power of the plantar flexors translate into a more symmetric translation of the body system as a whole (i.e., of the CoM)? Despite the recent shift of interest from purely kinematic to dynamic analyses at the level of the lower limb, energy changes of the CoM during split-belt walking have not been directly addressed to date. Evaluation of this factor might clarify whether the primary aim of late adaptation is the minimization of W_ext_ and/or of the plantar flexors' power and how these are achieved. Unfortunately, the time courses of E_tot_ and R during split gait have not yet been described.

### A Visible CoM Used to Steer Gait Control

There is only one report that describes an experiment in which the subject is given visual feedback of the oscillations of the CoM in the frontal plane during treadmill walking (124). This was achieved by projecting the vertical motion of a reflective marker placed on the sacrum onto a screen in front of the subject who is facilitated by “knowledge of results” (125). This enhanced feedback stimulates the search for an implicit neuro-mechanical solution. After a short series of rehabilitation sessions, the symmetry of the vertical CoM motion in hemiplegic subjects, analyzed with the double-integration method, was improved, and the metabolic cost of walking decreased. However, these isolated results are still awaiting independent confirmation.

## Discussion. The CoM Motion From Bench to Bedside: a Bumpy Road

Gait analysis is still striving to become a routine method of assessment of walking impairments. This goal is far from being achieved despite continuous educational efforts by dedicated scientific societies (e.g., SIAMOC in Italy, ESMAC at a European level).

### Instrumental Gait Analysis: Why Not Yet a Routine Clinical Approach?

The main obstacles to clinical applications of gait analysis, in the authors' opinions, are neither the cost nor the technical skills required. Many other diagnostic technologies common in medicine are complicated and expensive (e.g., those applied to imaging and neurophysiology). The key point is that, in most clinical conditions, gait analysis seems to add little to a skilled visual assessment, with respect to conventional clinical decision making (126). By greater force, the analysis of the CoM, not yet considered as a standard component of gait analysis, seems doomed to remain confined in the research domain. This is a particular case of a more general problem. Energy, work and power (three related physical variables) look abstract concepts to most clinicians, who are much more familiar with tangible muscle forces and, of course, with kinematics and visible electromyographic tracings. Abstract, however, here does equal neither imaginary nor non-existent. Forces alone do not explain the movement, which implies displacement in a given time, hence power (the product of force time shortening velocity). The neural sequences of muscle activation follow the logic of optimizing the power requirements and are adapted to mechanical requirements. Walking as a movement makes no exception. Its peculiarity lies in the skillful interplay between muscle power and gravity, culminating in the extremely advantageous pendulum-like mechanism, which is the result of a long biological evolution. It is clear, however, that any changes in the engines (the muscles), and/or in their neural control, and/or in the machinery (the joints) do have an impact on the motion of the body system as a whole. The many degrees of freedom of the human body, however, makes the type and amount of this impact hard to predict. In some cases, adaptation will aim at minimizing energy expenditure, hence the overall efficiency of the pendulum mechanism per unit distance. Sometimes the sparing of affected segments will be privileged, and in other cases, the best trade-off will be looked for between segment and system needs. In any case, the sources of kinematic impairments (will they be at a single joint or at the system level in claudication) can only be found in the changes of the patterns of power production.

### Neglecting the CoM Contributes to Make Gait Analysis Marginal

Having said that, the issues of the low clinical success of standard gait analysis based on segmental motions and the neglect toward CoM motion, look related. In the authors' opinion, the available technologies for the analysis of segmental kinematic, electromyographic, and dynamic events are informative and within reach, both technically and financially, of most in- and out-patient services. An international collaboration on the topic is very intense. The analysis of the CoM motion does not raise any insurmountable difficulties. When realized through the indirect kinematic method, studying the CoM motion only requires a dedicated software routine to be added to the software package for kinematic analysis provided by the manufacturers. The weak points remain the clinical questions, not the technical answers. The bulk of data emerging from a contemporary gait analysis should not be explored within a descriptive logic, which can turn into a fishing expedition with little chance of success. Rather, *a priori* hypotheses should be conceived, focused questions should be raised, and the analysis should be run according to a confirmatory (or, dis-confirmatory) logic, as one would for any diagnostic procedure. Unfortunately, walking in general and the CoM motion in particular are rarely considered as specific, trans-disciplinary chapters of medical education. By contrast, understanding the CoM motion might help to interpret the focal alterations. First, the CoM cannot be directly observed, but signs of alterations of its path can be visually detected (see Note S6). The clinical questions to be raised to gait analysis thus become (a) which are the segmental motions related to this “system” impairments? and (b) will any correction of these focal impairments through exercise, drugs, orthoses, or surgery lead to a more physiologic motion of the body system? Second, extremely simple and affordable techniques, such as those based on wearable accelerometers, might provide a rough quantitative analysis of the CoM motion, sufficient to help monitoring progress during a rehabilitation program in any clinical environment.

## Conclusions and Perspectives

Complete understanding of human walking will be facilitated if the issue is considered within the wider perspective of legged terrestrial locomotion.

### Legged Locomotion in Biology: Hints to the Clinical Interpretation of Walking Impairments (and of Their Resistance to Rehabilitation)

All walking animals fight against both gravity and ground shearing forces. In this context, it appears that the critical function of locomotion is made possible by mechanical strategies that are widespread within the animal realm. Oscillating legs are compatible with the nerve and vascular supply to the legs, and thus with life, in a way that rotational appendages, in the manner of wheels, would not be. Unlike wheeled locomotion, however, this solution entails costly repositioning of the limbs at every step, and costly upward and forward accelerations of the CoM at each cycle. The latter are the main causes of muscle work at low and intermediate walking velocities. It is not surprising that the inverted pendulum compromise, which reduces the otherwise exaggerated energetic cost of transport of the body system, is observed in the majority of walking species, despite their huge variety of sizes, shapes, locomotory structures and nervous systems. Among apes, human walking is special, as far as it is four times more efficient than either the quadrupedal or bipedal walking of Chimpanzees (127). This partly explains the capacity of the modern *Homo* genus to spread across the whole world during the “great human expansion” outside of Africa, from about 60,000 to 12,000 years B.C. (128). Given the vital role of walking and the extreme efficiency of the human pendulum-like mechanism, it is not surprising that humans tenaciously keep their CoM motion mechanism substantially unchanged from 4 years of age to senility, despite a trend toward joint degeneration and muscle weakening. Again unsurprisingly, the efficiency of CoM motion per unit distance, and often, per single step, is spontaneously preserved in the majority of human motor impairments: this explains their resistance to therapeutic attempts aiming to modify an otherwise successful and overwhelming adaptation strategy.

### Neural Gait Control Is Always Targeting the Invisible CoM

These considerations seem to support the conclusion that the motion of the CoM itself, not less than the motions of anatomical segments, is a constant target of neural control and should also be a primary target of clinical diagnostics. Segmental alterations should be suspected to be both causes and effects of alterations of CoM motion. If one lower limb has an altered motion, the rest of the body system will strive to adapt in order to optimize the balance between sparing the affected segment and maintaining the highest possible efficiency and safety of the pendular motion of the CoM. Relating the CoM motion to the segmental perspectives will allow original impairments to be distinguished from adaptive alterations.

### Future Directions, Between Adaptation and Restoration

Alterations of the 3D CoM pathway may be a sensitive index of pathology yet, there are few specifics with respect to segmental alterations: diagnosis will thus require clinical diagnostic skills. Therapeutic options will increase if clinicians consider walking from the perspective of the CoM motion, too. In symmetric pathological gaits, increasing velocity and step length (however obtained) may be rewarded by a more efficient pendulum mechanism. In asymmetric gaits the CoM perspective is particularly enlightening. On one hand, it seems clear that any attempt to restore symmetry by treating the affected side will be at high risk for failure, as the successful adaptation of CoM motion will be jeopardized. On the other hand, asymmetry leads to troubles in the long run, so that treatments that aim to rebalance the work between the affected and unaffected lower limbs are justified. These treatments need a conceptual advance in rehabilitation medicine and should be driven by monitoring the effects on CoM motion. The idea of forced-use through spilt-belt treadmills looks a promising paradigm of a system-and-segments approach for the diagnosis and treatment of asymmetric walking impairments. Other approaches that directly target the CoM, cited above, might be effective as well. As a final speculation, going perhaps beyond the scope of the present review, the potential role of non-invasive electric or magnetic brain stimulation can be alluded to. A variant (and perhaps one of the neurophysiologic bases) of the “acquired non-use” model is represented by the model of interhemispheric inhibition. Evidence is growing that the preference for an asymmetric locomotion is reflected by the asymmetry in excitability between the two hemispheric motor cortices, both in orthopedic and neurological impairments (110, 129, 130). No matter whether brain functional asymmetries are effects or causes of walking mechanical asymmetries, combining neurophysiological and behavioral treatments might make sense in the near future.

## Author Contributions

LT and VR discussed and contributed to writing the article. LT designed and wrote the content of the manuscript. All authors read and approved the final manuscript.

### Conflict of Interest Statement

The authors declare that the research was conducted in the absence of any commercial or financial relationships that could be construed as a potential conflict of interest.

## Supplementary Material

Video S1 Video showing a healthy subject walking on a split belt treadmill. Further details are provided in the video.
