# Supplementary material for: The Motion of Body Center of Mass During Walking: A Review Oriented to Clinical Applications
Source: Front Neurol. 2019 Sep 20;10:999. doi: 10.3389/fneur.2019.00999 (PMC6763727; doi:10.3389/fneur.2019.00999)
Supplement: Supplementary file 1 [file Table_1.docx]

**Note S1. More on the Center of Mass**

In generalizing the Newtonian mechanical theory from a single particle to a system composed of many particles, a distinction is introduced between the **internal** forces acting on the *i*th particle due to other particles in the system, and the **external** forces acting on the system due to other, outside-the-system sources. Using this decomposition, the equation of motion, Newton's second law, for the *i*th particle belonging to the system is given by:

$\dot{\boldsymbol{p}_{\boldsymbol{i}}}=\sum_{j} \boldsymbol{F}_{\boldsymbol{ij}}+\boldsymbol{F}_{i}^{ext}$ (1)

where $\boldsymbol{p}=m_{i}\dot{\boldsymbol{r}_{\boldsymbol{i}}}$ denotes the linear momentum, $\boldsymbol{F}_{ij}$ the (internal) force exercised by particle *j* on particle *i*, and $\boldsymbol{F}_{ij}^{ext}$ the external force on the *i*th particle.

Summing up all the particles in the system one reaches the following equation of motion for the system:

$\frac{d^{2}}{{dt}^{2}}\sum_{i} m_{i}\boldsymbol{r}_{\boldsymbol{i}}=\sum_{ij} \boldsymbol{F}_{\boldsymbol{ij}}+\sum_{i} \boldsymbol{F}_{\boldsymbol{i}}^{\boldsymbol{ext}}\equiv\boldsymbol{F}^{ext}$ (2)

where the sum over the internal forces $\boldsymbol{F}_{ij}$ vanishes because of Netwon’s third law of motion: the force two particles exert on each other is equal and opposite. Moreover, the force applied by a particle on itself is null, $\boldsymbol{F}_{ii}=0$, so that the sum may be taken over the indexes *i*, *j* such that *i ≠ j.*  Given the net external force on the system $\boldsymbol{F}^{ext}$, the center of mass of the system is defined as the average of the radii vectors of the system’s particles weighted over their masses:

$\boldsymbol{R=}\frac{\sum_{i} m_{i}\boldsymbol{r}_{i}}{\sum_{i} m_{i}}\boldsymbol{\equiv}\frac{\sum_{i} m_{i}\boldsymbol{r}_{i}}{M}$ (3)

where $M= \sum_{i} m_{i}$ is the total mass of the system. Using the definition of the center of mass above, the equation of motion for the system reads

$\frac{d^{2}\boldsymbol{R}}{{dt}^{2}}=\boldsymbol{F}^{ext}$ (4)

which is Newton’s equation in its canonical form for a single particle of mass *M*, described by a radius vector which coincides with the center of mass ***R***. Otherwise stated, the center of mass moves as if the net external force were acting on the entire mass of the system concentrated in the center of mass. Internal forces, due to Newton’s third law of motion, have no effect on the center of mass dynamics. For a detailed description of the topic see (Goldstein et al., 2002).

Goldstein, H., Poole, C., and Safko, J. (2002). *Classical Mechanics*. 3rd ed. Pearson Education Ltd.
