# Supplementary material for: The Motion of Body Center of Mass During Walking: A Review Oriented to Clinical Applications
Source: Front Neurol. 2019 Sep 20;10:999. doi: 10.3389/fneur.2019.00999 (PMC6763727; doi:10.3389/fneur.2019.00999)
Supplement: Supplementary file 2 [file Table_2.docx]

**Note S2. Methods for analyzing the motion of the CoM during walking**

This Note gives an overview of the most relevant solutions adopted, both theoretical and technical, to measure the CoM displacements, and the underlying changes of mechanical energy, during walking.

1. The direct, double-integration method

i. Physical principles

The displacements of the CoM, now within reach of scientific observation, are sustained by two sources of work and power, i.e. gravity and muscle contraction. The CoM motion, therefore, cannot be fully understood unless the underlying changes of mechanical energy are measured. The gold standard measurement for the motion of the CoM during walking is the ingenious method that was introduced by Cavagna (Cavagna, 1975). In this method, the motion of the CoM was originally analyzed in the sagittal plane only. The subject performs at least one entire stride at a steady average velocity, with both feet on a force platform that is sensitive to vertical and anteroposterior forces, which are easily recorded and analyzed nowadays by computerized systems. At each time point, an infinite number of forces (ground reactions) arise below the foot surface. The CoM translations, which are relevant for this study, are determined by the vectorially resultant total force. Rotations around the CoM additionally arise whenever the resultant force does not act on the CoM. Ground reaction forces are the products of a constant body mass and its variable accelerations. Their integration gives rise to changes in the forward and vertical velocities of the CoM, and hence, changes in kinetic energy in both directions (E_kf_ and E_kv_, respectively). Integration of the vertical velocity gives the vertical displacements, and hence, changes in gravitational potential energy, E_p_. Three constants must be known to evaluate the changes of energy in the sagittal plane: the average forward and vertical velocities and the subject’s mass. Furthermore, absolute velocity (measured from outside the platforms) enters the formula of kinetic energy. The value of E_kf_ is defined by Equation 1:

$\text{E}_{\text{kf}}\text{=}\frac{\text{1}}{\text{2}}\text{*m*}\text{V}_{\text{f}}^{\text{2}}$ (1)

Where m is the subject’s mass and V_f_ the instantaneous forward velocity. For calculation of E_kv_, the instantaneous vertical velocity is considered.

The subject’s weight must be zeroed before integration of vertical ground reaction forces (as opposing gravity does not lead to any velocity change; nor, therefore, to any displacement). Fig. 5 (Cavagna et al., 2000) illustrates these algorithms. The subtle technicalities of experimental recordings are described in several articles (Cavagna et al., 1983a; Willems et al., 1995).

**
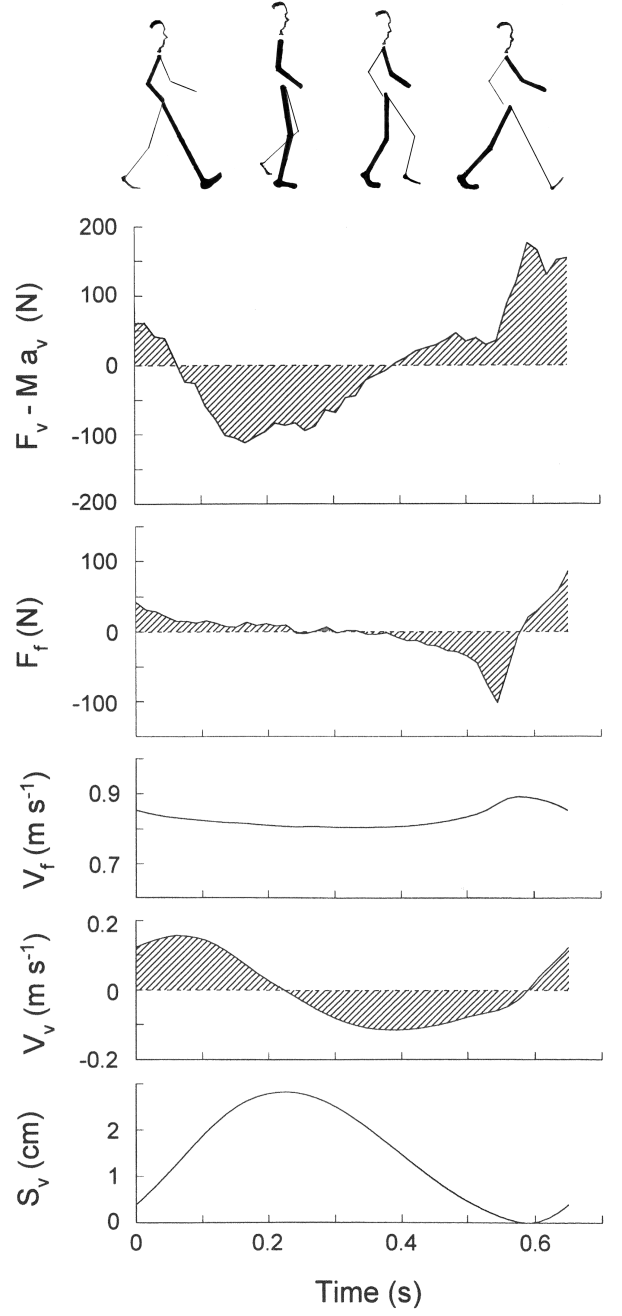
**

**Figure S2-1:** The principles of the double-integration method, also known as Cavagna’s or the Newtonian method, for computation of vertical displacement of the body center of mass (CoM) are shown in the context of one step. The step begins when the forward velocity hence, the kinetic energy, reaches a maximum. In real experiments the subject (sketched on top) performs entire strides at constant average velocity along a straight path on a long force-sensing platform. From top to bottom, the first panel gives subject mass (M) times the gravitational acceleration (a_v_) is subtracted from the vertical force (F_v_) so that the signal above and below zero is proportional to the net vertical acceleration of the subject’s CoM. The second panel gives the fore-aft force (F_f_) in this passage is positive when the force against the platform is directed forward causing deceleration of the CoM. The third panel gives the forward velocity (V_f_) obtained from the integration of F_f_ (hatched areas under the F_f_ curve), added to the constant of integration given by the average V_f_ measured using photocells. Lateral forces were not available in the original studies based on the double-integration method. The fourth panel gives the vertical velocity (V_v_) obtained from integration of the vertical force minus M (hatched areas under the F_v_−M*a_v_ curve) assuming the integration constant (i.e., average V_v_) is equal to zero. The last panel gives the vertical displacement of the CoM obtained from the integration, hence the double integration definition of the method, of the V_v_ (hatched areas under the V_v_ curve). Once the subject’s mass and velocities are known, changes in kinetic energy (½M*V^2^) can be computed. Once the vertical displacement (S_v_) is known, the gravitational potential energy (M*S_v_) can be computed. Adapted from Cavagna et al. (2000), used with permission.

In healthy subjects, the sagittal motion of the CoM is highly symmetric between the two steps within a stride (Cavagna et al., 1983b), although this may not be the case in pathological gaits (discussed below). Therefore, representation of at least two subsequent steps (one stride) has become a common approach in the clinical literature (Tesio et al., 1985). More recently, changes in the kinetic energy of the CoM laterally in the frontal plane (E_kl_) could be measured, due to the development of platforms that are sensitive to lateral forces (Tesio et al., 1985). The changes in E_kl_ are much smaller than those observed in the sagittal and vertical directions because lateral displacements and forces are smaller (Fig. 2 in the main text). The increments of E_tot_ represent the positive work necessarily done by muscles to keep the CoM in motion with respect to the ground (“external” work, W_ext_).

ii. Criticisms and limitations

ii.a. A critizable criticism: external work should be neglected when legs work “one agaist the other”

Some have argued that this method underestimates the changes of E_tot_ (hence, of W_ext_) during double stance. The objections, perhaps simplistically, can be summarized as follows. Classical studies of work during walking make a distinction between W_ext_, the positive work done by muscles to move the CoM with respect to the ground, and W_int_, the positive work done to move the body segments with respect to the CoM (Cavagna and Kaneko, 1977) (see also Supplementary Note S3). The double stance phase of walking, however, also allows for work by one leg (doing positive work) against the other (doing negative work). How can we best classify and measure this work? The literature on the topic only considers the sagittal plane. According to the so-called “individual legs model”, as opposed to the Newtonian “combined legs model”(Donelan et al., 2002), both the positive and negative work done by each leg during the double stance should be considered as external, i.e. applied to the CoM. The reason for this is that there can be no energy transfer between the two legs because there are no muscles that cross from one leg to the other. Across the range of velocities considered by the authors (0.5 m s^-1^ to 2.0 m s^-1^), the underestimation of positive and negative external work during the double stance should amount to about 33% (Donelan et al., 2002, their Fig. 5). To sum up, neglect of this external work should lead to underestimation of muscle work, thus resulting in errors in countless studies on external work based on the computation of the resultant ground force vectors (see next paragraph for further details).

The individual limbs model itself, however, has been thoroughly criticized on both a) theoretical and b) empiric grounds (Bastien et al., 2003). a) Summating the work done by individual limbs during double stance leads to an overestimation of their muscular work. First, a passive transfer occurs between the lost “vertical” energy (gravitational potential energy + kinetic energy due to the vertical speed) of the CoM, which falls within the first half of the double stance, and the work due to forward (or backward) forces applied by each leg, as per the inverted pendulum model of walking. Second, a mechanical energy transfer is indeed possible between two actuators, such as the legs, attached to the same mass provided they are not held orthogonally to each other. Third, one can assume that the legs work each against the other and not on the CoM, only when positive work is provided in the forward direction by the rear pushing leg, and negative work is simultaneously provided along the same direction by the front braking leg. The vertical component of work is external by definition, as within-limb motions are neglected in the individual limb model, as far as it acts by raising the CoM; b) The time course of the limbs’ work changes with the walking velocity. At low velocities, say, below 1 m s^-1^, entailing a short step length, during the double stance the front leg oscillates close to the vertical so that it exerts a modest braking action and the push from the trailing leg is almost entirely spent to raise the CoM. At higher speeds, say, above 1.5 m s^-1^, the forward push from the trailing leg tends to occur before the front leg strikes the ground, thanks to a forceful plantar flexion, which can be only spent to accelerate the CoM forward. These observations apply both to children older than four years and adults, once velocity is size-adjusted through the Froude number (Bastien et al., 2003). A bilateral contribution to W_ext_ during double stance is relevant only at intermediate velocities, approximately between 1 m s^-1^ and 1.5 m s^-1^. Above 11 years of age, this contribution peaks at velocities around 1.4 m s^-1^, and it can reach about 30% of Wext. To sum up, according to these criticisms, which the Auhtors of the present review endorse, the double integration method is correct in estimating W_ext_. It already takes into account all sources of external work, including the one done by “one leg against the other”. It remains true that the latter contributes to both external and internal work and that the current methods of measurement of W_int_ (not of W_ext_) underestimate, to an extent yet to be determined, Wint. This underestimation exists whichever the assumptions underlying the W_int_ estimation: transfer of energy across limb segments, transfer of energy between the limbs and the CoM, or no transfer at all (Willems et al., 1995).

ii.b. Limitations really intrinsic to the double integration method

The double-integration method has a theoretical limitation in that it can provide the CoM displacements with respect to an initial position, but it cannot locate the CoM within the body system nor with respect to the ground frame. It also has practical limitations, as it requires long and expensive platforms. At least one full stride must be performed on force sensors (Cavagna, 1975). The development of 3D force-sensing treadmills brought an exciting advancement to the method (Kram et al., 2017; Tesio and Rota, 2008), the technicalities of which are summarized in a user-friendly paper (Aarts et al., 2018). Force sensors are placed under a treadmill, or under each belt in the case of a treadmill with two parallel belts (split-belt treadmill, see below). A dedicated Note (Supplementary Note S3) highlights the limits and the outweighing advantages of the treadmill context.

In the case of both ground and treadmill walking, the double-integration method, based on force signals, faces practical challenges. Substantial limitations may affect the force sensors. These may be represented by metal strain-gauges or by piezoelectric quartz crystals. These two types of transducers present, among others, with a critical difference. Platforms based on quartz transducers have a higher dynamic sensitivity compared to strain-gauges platforms (resonant frequencies in the order of 800 *vs* 80 Hz, respectively). However, the quartz response to low frequency or constant loads is much less stable. The baseline force signal is drifting so that a continuously, non-linear offset develops until the system is reset, as correctly outlined by some authors (Bonnet et al., 2015). Given that forces undergo a double integration, the signal drift may lead to relevant errors in the computation of energy changes of the CoM, the higher the forces acting on the transducers, in particular, the vertical forces. This drift may limit the observation of walking to a maximum of a few minutes (Tesio and Rota, 2008) unless the error is empirically corrected on-line through high-pass filtering or off-line through algebraic manipulation of results. In most applications, however, a few tenths of strides are recorded in each run, which requires a handful of seconds.

1. Indirect methods: the inverse-dynamics and inertial sensors methods

The inverse-dynamics method can locate the CoM within the body system, and it eliminates the need for force sensors. Recording the displacements of numerous body landmarks during walking (e.g. from lower limb joints) is an affordable task using contemporary optoelectronic instruments. By reference to anthropometric tables, the inertia moments of body segments around the joints can be estimated. Motion capture methods enable calculation of velocity changes, allowing energy changes of body segments and, ultimately, of the CoM to be estimated (Faber et al., 2018). This method does have some theoretical and practical limitations. It only provides estimates, not a direct measurement, of the CoM motion (Cappozzo et al., 2005). However, studies performed on healthy subjects have compared the direct and indirect methods and several inverse dynamics models and have demonstrated that their results can be very similar, although not fully equivalent, with respect to the external work done directly on the CoM (Rao et al., 2006; Bonnet et al., 2015; Camomilla et al., 2017; Pavei et al., 2017). Another limitation of the indirect method is the requirement for expensive optoelectronic systems and accurate placement of reflective markers on many body landmarks according to anatomical models. Furthermore, a series of exhaustive reviews have summarized the instrumental errors (Chiari et al., 2005) as well as errors due to soft tissue artifacts (Leardini et al., 2005) and marker misplacement (Della Croce et al., 2005).

Some authors have adopted the “individual limb method” (see previoos paragraph) which is a hybrid non-Newtonian method. To compute CoM energy changes the ground reaction forces under each foot are measured experimentally, but the CoM velocity is estimated through the double integration method or from optical-anatomical modeling. The power applied to the CoM during double stance is calculated as the integral of the forces under every single foot, multiplied by the velocity of the CoM. This method results in higher estimates of W_ext_ during the double stance period of the stride, compared with the double-integration method (Donelan et al., 2000; Donelan et al., 2002; Neptune et al., 2004). Although this method was adopted by some researchers (Wurdeman et al., 2013; Zelik and Adamczyk, 2016; Selgrade et al., 2017), its theoretical superiority compared with the double-integration method has been severely criticized (Bastien et al., 2003) (see previous paragraph). Of special interest here, whichever its amount, this is internal and not external work.

Other indirect methods assume that the sacrum can be taken as a proxy of the location of the CoM within the body system. This leads to a rough approximation, given that the CoM can move with respect to the sacrum. Analysis based on a sacral inertial marker, a single optical sacral marker or a simplified optical markers’ set (Eames et al., 1999; Floor-Westerdijk et al., 2012; Gard et al., 2004; Thirunarayan et al., 1996; Tisserand et al., 2016; Yang and Pai, 2014) were tested in healthy and impaired adults and children, and compared with results obtained from established standards, i.e. the force-plate method and refined kinematic models. Albeit of some usefulness for simple analyses in the absence of refined equipment, these methods are less reliable, mostly when applied to measures of lateral displacements of the CoM at high walking velocities and pathologic gaits.

Other theoretical approaches exist for estimating the location of the body CoM. A substantial reduction of optic markers might be achieved by refining the conventional method based on anthropometric modeling of body segments and the application of numerous markers (in the order of several tenths) (Tisserand et al., 2016) or by ascribing masses to a very few body markers (Forsell and Halvorsen, 2009). After an optical and dynamometric calibration in selected static postures, modelling based on multilink branched joint chains, a method coming from robotics research, allows estimation of the CoM position with no further dynamic assumptions or measures (Cotton et al., 2009; Bonnet et al., 2015). These approaches, to date, were mostly validated in static postures or athletic gestures.

Aarts, L., Papegaaij, S., Steenbrink, F., and Martens, P. (2018). “Quality of treadmill embedded force plates for gait analysis,” in *White Paper* (Motek).

Bastien, G. J., Heglund, N., and Schepens, B. (2003). The double contact phase in walking children. *J. Exp. Biol.* 206, 2967–2978. doi:10.1242/jeb.00494.

Bonnet, V., González, A., Azevedo-Coste, C., Hayashibe, M., Cotton, S., and Fraisse, P. (2015). Determination of subject specific whole-body centre of mass using the 3D statically equivalent serial chain. *Gait Posture* 41, 70–75. doi:10.1016/j.gaitpost.2014.08.017.

Camomilla, V., Cereatti, A., Cutti, A. G., Fantozzi, S., Stagni, R., and Vannozzi, G. (2017). Methodological factors affecting joint moments estimation in clinical gait analysis: A systematic review. *Biomed. Eng. Online* 16, 1–27. doi:10.1186/s12938-017-0396-x.

Cappozzo, A., Della Croce, U., Leardini, A., and Chiari, L. (2005). Human movement analysis using stereophotogrammetry: Part 1: theoretical background. *Gait Posture* 21, 186–196. doi:10.1016/j.gaitpost.2004.01.010.

Cavagna, G. A. (1975). Force platforms as ergometers. *J. Appl. Physiol.* 39, 174–179. doi:10.1093/icb/40.1.101.

Cavagna, G. A., Franzetti, P., and Fuchimoto, T. (1983a). The mechanics of walking in children. *J. Physiol.* 343, 323–339. doi:10.1113/jphysiol.1983.sp014895.

Cavagna, G. A., and Kaneko, M. (1977). Mechanical work and efficiency in level walking and running. *J. Physiol.* 268, 467–481.

Cavagna, G. A., Tesio, L., Fuchimoto, T., and Heglund, N. C. (1983b). Ergometric evaluation of pathological gait. *J. Appl. Physiol.* 55, 607–613. doi:10.1152/jappl.1983.55.2.606.

Cavagna, G. A., Willems, P. A., and Heglund, N. C. (2000). The role of gravity in human walking: Pendular energy exchange, external work and optimal speed. *J. Physiol.* 528, 657–668. doi:10.1111/j.1469-7793.2000.00657.x.

Chiari, L., Della Croce, U., Leardini, A., and Cappozzo, A. (2005). Human movement analysis using stereophotogrammetry. Part 2: Instrumental errors. *Gait Posture* 21, 197–211. doi:10.1016/j.gaitpost.2004.04.004.

Cotton, S., Murray, A. P., and Fraisse, P. (2009). Estimation of the center of mass: from humanoid robots to human beings. *IEEE Trans. Mechatronics* 14, 707–712. doi:10.1109/TMECH.2009.2032687.

Della Croce, U., Leardini, A., Chiari, L., and Cappozzo, A. (2005). Human movement analysis using stereophotogrammetry Part 4: Assessment of anatomical landmark misplacement and its effects on joint kinematics. *Gait Posture* 21, 226–237. doi:10.1016/j.gaitpost.2004.05.003.

Donelan, J. M., Kram, R., and Kuo, A. D. (2002). Simultaneous positive and negative external mechanical work in human walking. *J. Biomech.* 35, 117–24. Available at: http://www.ncbi.nlm.nih.gov/pubmed/11747890 [Accessed September 18, 2018].

Eames, M. H. A., Cosgrove, A., and Baker, R. (1999). Comparing methods of estimating the total body centre of mass in three-dimensions in normal and pathological gaits. *Hum. Mov. Sci.* 18, 637–646. doi:10.1016/S0167-9457(99)00022-6.

Faber, H., Van Soest, A. J., and Kistemaker, D. A. (2018). Inverse dynamics of mechanical multibody systems: An improved algorithm that ensures consistency between kinematics and external forces. *PLoS One* 13, 1–16. doi:10.1371/journal.pone.0204575.

Floor-Westerdijk, M. J., Schepers, H. M., Veltink, P. H., Van Asseldonk, E. H. F., and Buurke, J. H. (2012). Use of inertial sensors for ambulatory assessment of center-of-mass displacements during walking. *IEEE Trans. Biomed. Eng.* 59, 2080–2084. doi:10.1109/TBME.2012.2197211.

Forsell, C., and Halvorsen, K. (2009). A method for determining minimal sets of markers for the estimation of center of mass, linear and angular momentum. *J. Biomech.* 42, 361–365. doi:10.1016/j.jbiomech.2008.10.029.

Gard, S. A., Miff, S. C., and Kuo, A. D. (2004). Comparison of kinematic and kinetic methods for computing the vertical motion of the body center of mass during walking. *Hum. Mov. Sci.* 22, 597–610. doi:10.1016/J.HUMOV.2003.11.002.

Kram, R., Griffin, T. M., Donelan, J. M., and Chang, Y. H. (2017). Force treadmill for measuring vertical and horizontal ground reaction forces. *J. Appl. Physiol.* 85, 764–769. doi:10.1152/jappl.1998.85.2.764.

Leardini, A., Chiari, L., Croce, U. Della, and Cappozzo, A. (2005). Human movement analysis using stereophotogrammetry: Part 3. Soft tissue artifact assessment and compensation. *Gait Posture* 21, 212–225. doi:10.1016/j.gaitpost.2004.05.002.

Pavei, G., Seminati, E., Cazzola, D., and Minetti, A. E. (2017). On the estimation accuracy of the 3D body center of mass trajectory during human locomotion: Inverse vs. forward dynamics. *Front. Physiol.* 8, 1–13. doi:10.3389/fphys.2017.00129.

Rao, G., Amarantini, D., Berton, E., and Favier, D. (2006). Influence of body segments’ parameters estimation models on inverse dynamics solutions during gait. *J. Biomech.* 39, 1531–1536. doi:10.1016/j.jbiomech.2005.04.014.

Selgrade, B. P., Thajchayapong, M., Lee, G. E., Toney, M. E., and Chang, Y. H. (2017). Changes in mechanical work during neural adaptation to asymmetric locomotion. *J. Exp. Biol.* 220, 2993–3000. doi:10.1242/jeb.149450.

Tesio, L., Civaschi, P., and Tessari, L. (1985). Motion of the center of gravity of the body in clinical evaluation of gait. *Am. J. Phys. Med.* 64, 57–70.

Tesio, L., and Rota, V. (2008). Gait analysis on split-belt force treadmills: validation of an instrument. *Am. J. Phys. Med. Rehabil.* 87, 515–526. doi:10.1097/PHM.0b013e31816f17e1.

Thirunarayan, M. A., Kerrigan, C., Rabuffetti, M., Croce, U. Della, and Saini, M. (1996). Comparison of three methods for estimating vertical displacement of center of mass during level walking in patients. *Gait Posture* 4, 306–314. doi:10.1016/0966-6362(95)01058-0.

Tisserand, R., Robert, T., Dumas, R., and Chèze, L. (2016). A simplified marker set to define the center of mass for stability analysis in dynamic situations. *Gait Posture* 48, 64–67. doi:10.1016/j.gaitpost.2016.04.032.

Willems, P. A., Cavagna, G. A., and Heglund, N. C. (1995). External, internal and total work in human locomotion. *J. Exp. Biol.* 198, 379–393.

Wurdeman, S. R., Huisinga, J. M., Filipi, M., and Stergiou, N. (2013). Multiple sclerosis alters the mechanical work performed on the body’s center of mass during gait. *J. Appl. Biomech.* 29, 435–42.

Yang, F., and Pai, Y.-C. (2014). Can sacral marker approximate center of mass during gait and slip-fall recovery among community-dwelling older adults. *J. Biomech.* 47, 3807–3812. doi:10.1016/j.jbiomech.2014.10.027.

Zelik, K. E., and Adamczyk, P. G. (2016). A unified perspective on ankle push-off in human walking. *J. Exp. Biol.* 219, 3676–3683. doi:10.1242/jeb.140376.
