# Supplementary material for: The Motion of Body Center of Mass During Walking: A Review Oriented to Clinical Applications
Source: Front Neurol. 2019 Sep 20;10:999. doi: 10.3389/fneur.2019.00999 (PMC6763727; doi:10.3389/fneur.2019.00999)
Supplement: Supplementary file 3 [file Table_3.docx]

**Note S3. Walking on the ground vs walking on a treadmill**

Whichever the method adopted for the analysis of the CoM motion, walking on a treadmill has several experimental advantages over walking on the ground. It allows for unlimited steps in a limited space, and it imposes a known and stable velocity, thus increasing the reliability of any measures. Treadmill walking speeds up experimental tests: a great benefit when patients or children are analyzed. Unless the belt slows down at heel strike, which is not the case with high-quality devices, the two forms of walking are dynamically equivalent (Willems and Gosseye, 2013). The treadmill length gives an obvious limitation. High walking velocities (say, above 1.4 m s^-1^) may lead to stepping outside the belt, in case of long strides and/or, in case the average subject's velocity does not match the treadmill's velocity. Differences in perceptual nature have been detected. Walking on treadmill implies an unusual sensory conflict: vision is signalling a stable position while proprioception is signalling forward motion, a partial natural solution to the conflict being provided by a visual illusion of motion (Yabe and Taga, 2008). Subtle behavioral consequences follow, all reflecting caution in facing the sensorimotor decoupling. The spontaneous average velocity adopted on the treadmill is lower by about 30% than the one adopted on firm ground (Rosenblatt and Grabiner, 2010). For any given velocity, step length is lower and, correspondingly, step frequency is higher, by about 8%; a slightly more flexed posture is adopted by hips and knees. These differences hold both for adults (Murray et al., 1985; Riley et al., 2007; Tesio and Rota, 2008) and children (Tesio et al., 2017).

Also, higher step width and a lower step width variability have been observed. Unexpectedly, the margin of stability, which is the distance between the CoM projection to the ground and the lateral side of the supporting foot (see main text), is unchanged (Rosenblatt and Grabiner, 2010). These observations suggest that a higher caution characterizes treadmill walking, leading to some compensatory adaptations. Treadmills have been suggested as a neuromotor therapeutic tool (see main text). Some raised the objection, supported by experimental findings, on stroke and spinal cord injured patients in robot-assisted tests, that the high task-specificity of treadmill walking may limit the learning of adaptive capacities to real-life environments (Mignardot et al., 2017).

Mignardot, J., Le Goff, C., van den Brand, R., Capogrosso, M., Fumeaux, N., Vallery, H., et al. (2017). A multidirectional gravity-assist algorithm that enhances locomotor control in patients with stroke or spinal cord injury. *Sci. Transl. Med.* 9, eaah3621. doi:10.1126/scitranslmed.aah3621.

Murray, M., Spurr, G., Sepic, S., Gradner, G., and Mollinger JA (1985). Treadmill vs. floor walking:kinematics, electromyogram, and heart rate. *J. Appl. Physiol.* 59, 87–91. doi:10.1152/jappl.1985.59.1.87.

Riley, P. O., Paolini, G., Della Croce, U., Paylo, K. W., and Kerrigan, D. C. (2007). A kinematic and kinetic comparison of overground and treadmill walking in healthy subjects. *Gait Posture* 26, 17–24. doi:10.1016/j.gaitpost.2006.07.003.

Rosenblatt, N. J., and Grabiner, M. D. (2010). Measures of frontal plane stability during treadmill and overground walking. *Gait Posture* 31, 380–384. doi:10.1016/j.gaitpost.2010.01.002.

Tesio, L., Malloggi, C., Portinaro, N. M., Catino, L., Lovecchio, N., and Rota, V. (2017). Gait analysis on force treadmill in children: comparison with results from ground-based force platforms. *Int. J. Rehabil. Res.* 40, 315–324. doi:10.1097/MRR.0000000000000243.

Tesio, L., and Rota, V. (2008). Gait analysis on split-belt force treadmills: validation of an instrument. *Am. J. Phys. Med. Rehabil.* 87, 515–526. doi:10.1097/PHM.0b013e31816f17e1.

Willems, P. A., and Gosseye, T. P. (2013). Does an instrumented treadmill correctly measure the ground reaction forces? *Biol. Open* 2, 1421–1424. doi:10.1242/bio.20136379.

Yabe, Y., and Taga, G. (2008). Treadmill locomotion captures visual perception of apparent motion. *Exp. Brain Res.* 191, 487–494. doi:10.1007/s00221-008-1541-3.
