# Supplementary material for: The Motion of Body Center of Mass During Walking: A Review Oriented to Clinical Applications
Source: Front Neurol. 2019 Sep 20;10:999. doi: 10.3389/fneur.2019.00999 (PMC6763727; doi:10.3389/fneur.2019.00999)
Supplement: Supplementary file 4 [file Table_4.docx]

**Note S4. Measurement parameters of the center of mass motion**

Whenever the work done by muscles to keep the CoM in motion (W_ext_) is calculated per single step or per unit distance (meters), it is dubbed W_ext,step_ or W_ext,m_ respectively. Notably, this positive work is necessarily provided by muscles that are undergoing active shortening; however, identifying which particular muscles are engaged is an intricate issue. Muscles also generate internal work, W_int_, which is the work required to move the body segments, in particular the limbs, with respect to the CoM, per step or unit distance. The total mechanical work done can be estimated by the sum of W_ext_ and W_int_. At low walking velocities (say, below 0.8 m s^-1^), W_int_ < W_ext_ (Cavagna and Kaneko, 1977). It is unlikely that W_int_ contributes substantially to the motion of the CoM, and the highest possible fraction of W_ext_ contributed by W_int_ is estimated to lie below 10% (Willems et al., 1995). For this reason, W_int_ is of limited relevance in the study of most pathological gaits, adopting low velocities. Research on W_ext_ has mostly focused on its changes as a function of average walking velocity (Cavagna et al., 1976), age, in the case of children (Cavagna et al., 1983; Dierick et al., 2004; Schepens and Detrembleur, 2009), and step frequencies for any given average velocity (Cavagna and Franzetti, 1986). External work per step in the vertical, forward, and lateral directions (W_v,step_, W_f,step_, and W_l,step_, respectively), which largely arise from passive energy exchanges, have also been investigated. Altogether, these studies clarified the importance of the pendulum mechanism over a step, which reflects the efficiency of mechanical energy transfer. This parameter was named % Recovery (R in the present review) (Cavagna et al., 1976). For instantaneous R and average R over one step, the notations R_inst_ and R_step_ will be adopted, respectively. R_step_ can be calculated using a simple formula (Equation 2):

$\text{R}_{\text{step}}\text{=}\frac{\text{W}_{\text{f,step}}\text{+}\text{W}_{\text{v,step}}\text{+}\text{W}_{\text{l,step}}\text{-}\text{W}_{\text{ext,step}}}{\text{W}_{\text{f,step}}\text{+}\text{W}_{\text{v,step}}\text{+}\text{W}_{\text{l,step}}}\text{*100}$ (1)

Initially, the step period was defined as the time interval between subsequent maxima of E_kf_. This convention led to the calculation that pendulum efficiency as an average over the whole step can reach 60% at velocities very close to that which is spontaneously adopted by human subjects (around 1.3 m s^-1^ or 4.7 km hr^-1^). This speed is also close to the velocity of minimum metabolic expenditure (estimated from oxygen consumption) and a minimum of W_ext_ per unit distance, although W_int_ also contributes (Cavagna and Kaneko, 1977). At velocities both higher and lower than the optimum, R_step_ is reduced, thus mirroring the U-shaped function (with reversed, downward concavity) of metabolic expenditure. Not surprisingly, most bipedal and quadrupedal animals prefer a walking velocity that maximizes R_step_ (Cavagna et al., 1977). At any given walking velocity, W_ext_ is influenced by the combination of step frequency (cadence) and length. A higher cadence (and so, a shorter step) is associated with lower braking forces from the ground, lower need for forward acceleration and deceleration of the CoM at foot strike, and smaller lift of the CoM during the subsequent oscillation on a single lower limb. When measured per unit time, increasing the cadence decreases the external work while the internal work increases. Again, for any given velocity, the maximum R_step_ and minimum E_tot_ values occur at a cadence close to the self-selected one (Cavagna and Franzetti, 1986). This cadence can be sustained to minimize the positive total external work produced both per step and per unit distance, which is one of the main constraints of walking mechanics.

Cavagna, G. A., and Franzetti, P. (1986). The determinants of the step frequency in walking in humans. *J. Physiol.* 373, 235–242. doi:10.1113/jphysiol.1986.sp016044.

Cavagna, G. A., Franzetti, P., and Fuchimoto, T. (1983). The mechanics of walking in children. *J. Physiol.* 343, 323–339. doi:10.1113/jphysiol.1983.sp014895.

Cavagna, G. A., Heglund, N., and Taylor, C. (1977). Mechanical work in terrestrial locomotion: two basic mechanisms for minimizing energy expenditure. *Am. J. Physiol.* 233, 243–261. doi:https://doi.org/10.1152/ajpregu.1977.233.5.R243.

Cavagna, G. A., and Kaneko, M. (1977). Mechanical work and efficiency in level walking and running. *J. Physiol.* 268, 467–481.

Cavagna, G. A., Thys, H., and Zamboni, A. (1976). The sources of external work in level walking and running. *J. Physiol.* 262, 639–657.

Dierick, F., Lefebvre, C., Van Den Hecke, A., and Detrembleur, C. (2004). Development of displacement of centre of mass during independent walking in children. *Dev. Med. Child Neurol.* 46, 533–539. doi:10.1111/j.1469-8749.2004.tb01011.x.

Schepens, B., and Detrembleur, C. (2009). Calculation of the external work done during walking in very young children. *Eur. J. Appl. Physiol.* 107, 367–373. doi:10.1007/s00421-009-1132-4.

Willems, P. A., Cavagna, G. A., and Heglund, N. C. (1995). External, internal and total work in human locomotion. *J. Exp. Biol.* 198, 379–393.
