# Supplementary material for: The Motion of Body Center of Mass During Walking: A Review Oriented to Clinical Applications
Source: Front Neurol. 2019 Sep 20;10:999. doi: 10.3389/fneur.2019.00999 (PMC6763727; doi:10.3389/fneur.2019.00999)
Supplement: Supplementary file 5 [file Table_5.docx]

**Note S5. Pathological gaits: neither metabolic nor mechanical inefficiency is a mandatory rule**

Before the emergence of studies on the mechanics of CoM motion, numerous studies were conducted on total energy expenditure during gait, heralded by the seminal work of Rodolfo Margaria (Margaria, 1938). More recently, various pathological gaits were extensively investigated, and generally found to be inefficient in the sense that the energy expenditure defined as the mass-adjusted metabolic cost of gait, net of the standing cost, per unit distance also known as the cost of transport, or simply cost, was higher than normal (Waters and Mulroy, 1999). However, as counterintuitive as it may be, this seems not a mandatory rule. A common mistake in the literature has been to compare patients and control subjects at their spontaneous (comfortable, or self-selected) speeds. This approach ignores the U-shaped relationship of cost to velocity, reflecting the constraints of the pendulum oscillations. If the patients spontaneously walked at slower speeds than controls, a physiologic increase of cost would be expected independent of underlying impairment. The lower velocity itself could result in the differences in cost between patients and controls being nil or rather moderate; a suggestion that has been confirmed in a meta-analysis of data collected from post-stroke, lower-limb amputee, paraparetic, and healthy subjects (Tesio, 1991). Even severe, dynamically asymmetric gaits may not escape this rule. For instance, a recent study on transfemoral amputees could not find a significant relationship between the metabolic economy and both step period and length asymmetry (Mahon et al., 2019). In hemiparetic patients, both step length and period asymmetries were found to entail a lower cost compared to a forced symmetric gait, at whichever velocity (Roemmich et al., 2019). The above-cited review (Waters and Mulroy, 1999) analyzed the metabolic energy expenditure over the most various walking impairments: from ankle fusion to stroke, to paraplegia, and the most various orthotic adaptations: from shoe heels to crutches, to reciprocating gait orthoses. This study confirmed that a slower spontaneous velocity is the rule for impaired persons and that metabolic inefficiency is not a mandatory rule. However, it stressed that inefficiency is the most frequent finding. When comparisons were available with normal data taken at corresponding velocities (see the case of hemiplegic patients (Zamparo et al., 1995)), patients’ walking cost tended to be higher than normal, the more, the lower the velocity (usually reflecting more severe impairment). This finding suggested that the increase in energy expenditure per unit distance paralleled the severity of the motor impairment. For instance, it progressed from ankle fusion to knee immobilization, to hip fusion. The cost was normal in mild paraparetic subjects showing reciprocal crutch-assisted gait and was highly increased in those adopting a more demanding swing-through crutch-assisted gait. As a partial explanation for these inconsistencies, it must be considered that spasticity (whichever its origin) entails an expensive agonist-antagonist co-activation which in itself generates energy expenditure but, in the absence of displacements, not work. A more recent study, however, confirmed earlier findings that hemiplegic gait presents with the above mentioned U-shaped cost-velocity relationship. This finding points again towards a normal efficiency, once velocity is taken into account (Reisman DS et al., 2009). These contradictory findings warn against generalization across impairments and individuals. Whichever its prevalence, the finding that at least some patients with asymmetric gaits may walk with a cost normal given the adopted velocity remains intriguing. In fact, in healthy subjects forcing step time asymmetry entails an increased cost of walking (Ellis et al., 2013).

The study of CoM motion has revitalized the debate on how normal metabolic efficiency is possible, at least in some asymmetric patients. From 1983 (Cavagna et al., 1983), the double-integration method was adapted to pathological gaits. A key focus of these studies was the symmetry of various parameters of CoM motion between the left and right steps. Symmetry is observed in healthy subjects, while asymmetry is characteristic of subjects with unilateral impairments. This finding was initially confirmed in subjects with post-stroke hemiparesis and unilateral hip arthritis (Tesio et al., 1985). Unexpectedly, during the step performed with single stance on the affected lower limb, W_ext,step_ was much lower, and R_step_ was much higher than in both the subsequent “healthy” step and the steps of healthy subjects walking at the same velocity. This mechanism suggests a reduction in work produced by the affected lower limb. Notably, the average values of W_ext_ and R from the two subsequent steps (i.e., per unit distance) were normal (Figure S5-1).


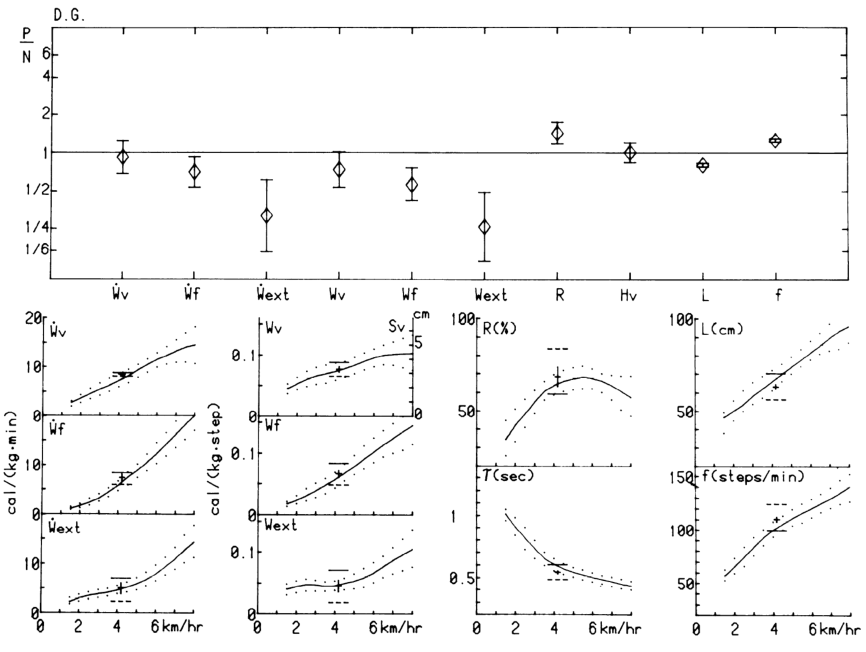


**FIGURE S5-1:** The upper panel shows the “index of asymmetry” of various parameters of the motion of the CoM between the pathologic (P) and normal (N) lower limbs in a representative patient affected by unilateral hip arthritis causing modest claudication. Walking was performed on force platforms embedded in the floor. The normal index equals 1 (Tesio et al., 1985). The P/N value is given, in log form, on the ordinate. Diamonds indicate the average index (± standard deviation) across 10 strides. The variables given on the abscissa are the external power output during a step ($\dot{w}$), the work per step (W), the percent recovery (R) per step, and the step period (τ), length (L), and frequency (f). For the $\dot{w}$ and W parameters, the –v and –f suffixes indicate the power and work required to lift the CoM and to accelerate it forward, while the –ext suffix indicates the external muscular work needed to keep the CoM in motion with respect to the ground, due to the incomplete transfer between its kinetic and gravitational potential energy. In the lower panels the same variables are given as a function of the average forward velocity. In the lower panels, continuous lines were traced by hand through the average of values measured on 17 normal subjects; dotted lines represent standard deviation. Crosses indicate the values for the patient analyzed in the uppermost panel, measured across a whole stride (cross center = mean, cross arms = standard deviation on both axes). Crosses are encased by a dashed and a continuous horizontal segment, referring to the mean values for the P and N step, respectively. Their ratio, P/N, is plotted in the uppermost panel. It can be seen that, when averaged between the P and N steps, values lie within normal limits (dotted curves). Taken from Cavagna et al. (1983b), used with permission.

The same finding has been confirmed in other forms of unilateral impairments. In prosthetised unilateral lower limb amputees, these asymmetries were found to be greater in above- compared with below-knee amputees (Tesio et al., 1998) (Figure S5-2).

**
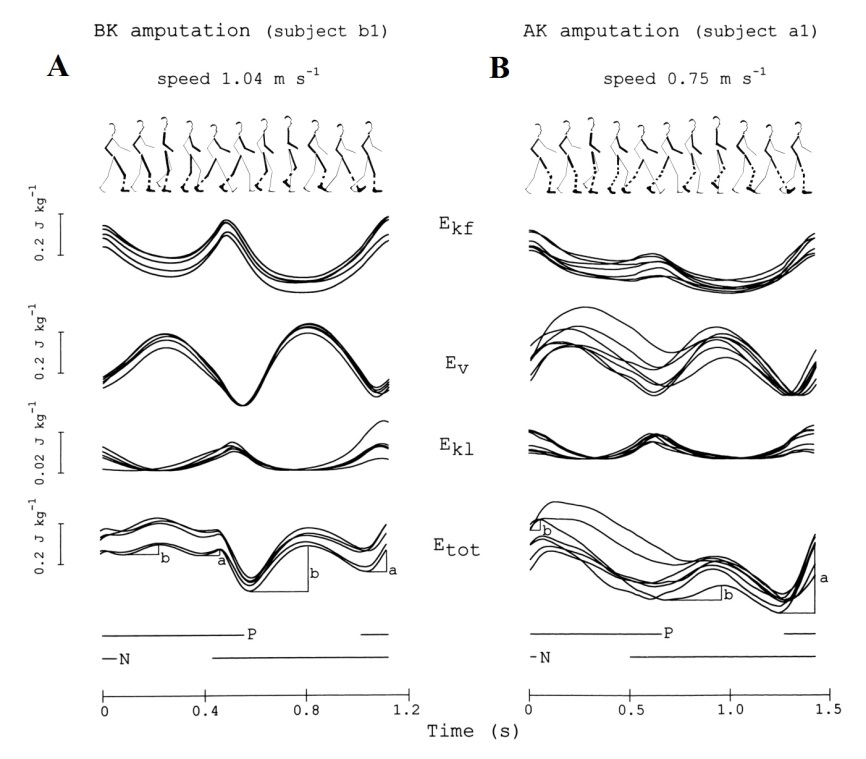
**

**FIGURE S5-2:** From top to bottom, the curves refer to the mechanical energy changes of the CoM due to the motion in the forward, vertical, and lateral directions (E_kf_, E_v_ and E_kl_, respectively) and their sum, E_tot_ = E_kf_ + E_v_ + E_kl_ (note the scale difference for E_kl_) as a function of time (normalized to stride duration). One entire stride (two subsequent steps), beginning with the step mainly performed over the prosthetized (P) limb, was analyzed in a transtibial prosthetized amputee (below-knee [BK], A panel) and in a transfemoral prosthetized amputee (above-knee [AK], B panel), sketched on top, walking at their preferred speed. Hatched tract: amputee lower limb. Each step is considered to begin when E_kf_ reaches a maximum (usually, while both feet are on ground). Curves from different strides, performed at about the same average speed (±10%), are superimposed. The bottom horizontal lines mark the time intervals in which the P or the normal (N) lower limb is on ground. During the double- and single-stance phases of the stride, increments of E_tot_ occur (a and b, respectively), which must be sustained by positive muscle work. In the amputee patients the increments of E_tot_ are very small and can hardly be distinguished from each other in the P step. The CoM oscillation over the amputated lower limb is virtually passive, like in an ideal inverted pendulum. The opposite occurs during the step sustained by the normal limb. The asymmetry is higher in the AK, compared to the BK, patient. Adapted from Tesio et al. (1998b), used with permission.

Again, when averaged along the entire stride, R and W_ext_ were normal (Figure S5-3).


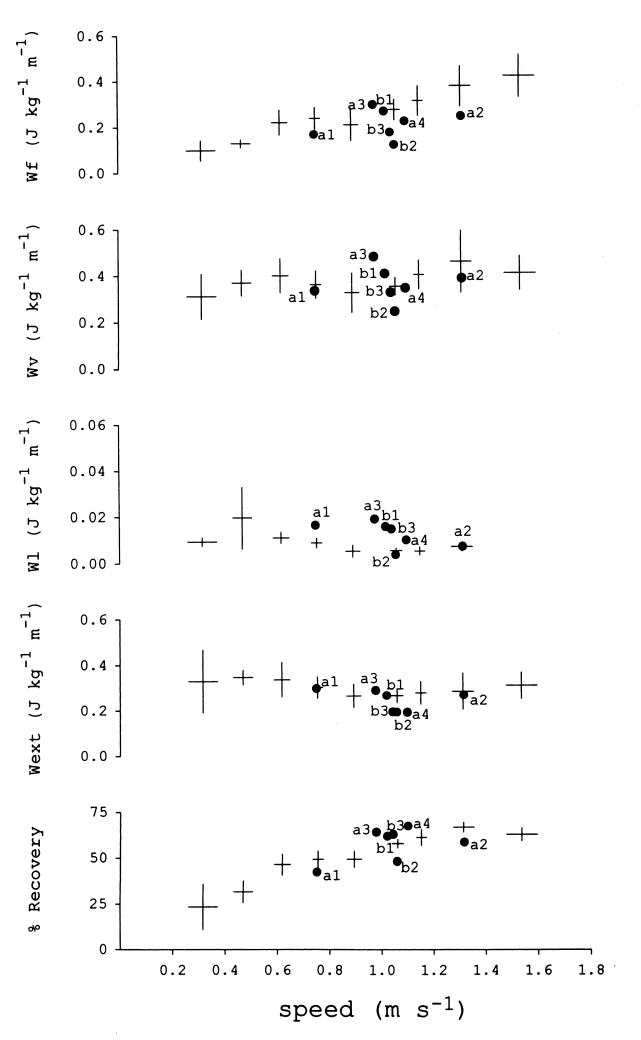


**FIGURE S5-3:** The four upper panels illustrate the positive work done per unit mass and distance (on the ordinate) to sustain the changes of mechanical energy of the center of mass (CoM) during walking as a function of the average forward velocity (on the abscissa). From top to bottom: W_f_, W_v_, and W_l_ (note the scale difference for W_l_) represent the work done to sustain the forward accelerations, vertical lifts and lateral accelerations of the CoM, respectively. W_ext_ is the muscular work done to sustain the increments of the total mechanical energy of the CoM (E_tot_, see Figure 3B). The bottom graph gives the amount of recovery (R) of mechanical energy. The crosses give the average values and 95% confidence limits in both axes, calculated in a previous work (Tesio et al. 1998a) from 125 steps performed by eight healthy subjects at various gait speeds. Each cross is centered over the mean speed (from 0.2 to 1.8 m s^-1^). Solid symbols indicate the mean values from the seven amputees (b1 to b3 = below-knee amputees; a1 to a4 = above-knee amputees). Taken from Tesio et al. (1998b), used with permission.

A normal W_ext,m_ despite relevant step asymmetries has been reported following a rare and complicated procedure of surgical limb-salvage, knee rotationplasty (Rota et al., 2016) (illustrated in Figure S5-4). In this operation, the lower thigh and upper leg are amputated, and the remaining leg is rotated by 180° and connected to the thigh stump. The ankle thus becomes a pseudo-knee requiring orthotic prolongation, and the soleus (gastrocnemii are disconnected) becomes a weak pseudo-quadriceps femoris. Interestingly, in line with the weakness of the pseudo-knee compared to normal conditions, the asymmetries in CoM motion following knee rotationplasty were found to lie between those observed in above- and below-knee amputees (Figure S5-4B) (Rota et al., 2016). Interestingly enough, the cortical motor map (built on motor-evoked potentials) of the rotated soleus shrinks, while the map of the unaffected soleus is enlarged (Tesio et al., 2014).

**
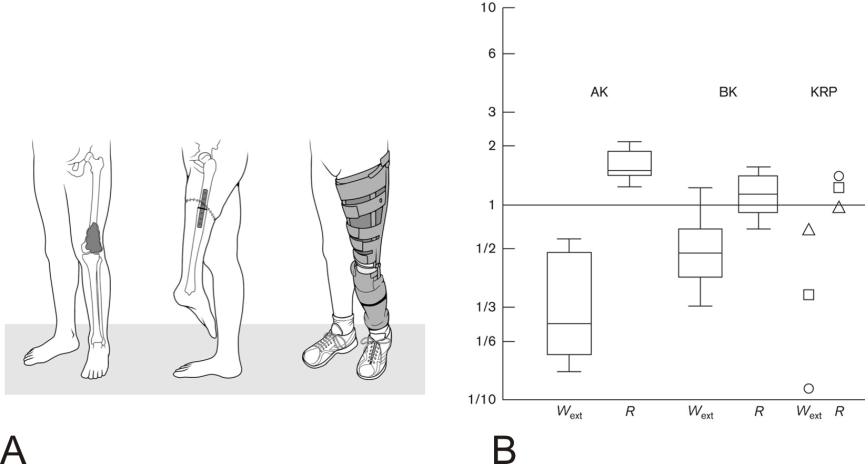
**

**FIGURE S5-4:** (A): Sketch of knee rotationplasty. From left to right: distal thigh tumor image, limb reconstruction and rotated leg image, and image of a patient wearing his/her prosthesis. Taken from Rota et al. (2016) used with permission. (B): Affected/unaffected (P/N) step ratio (log scale) of external muscular work (W_ext_) and recovery of muscle work (R) in (from left to right) above-knee amputees (AK), below-knee amputees (BK) and knee rotationplasty patients (KRP). In healthy individuals, the ratio is 1 (horizontal continuous line, Tesio et al., 1985) with minimal variation. The R ratios were 0.9, 1.3, and 1.4 in KRP patients (triangle, square, and circle symbols, respectively). Taken from Rota et al. (2016), used with permission. On average, R is higher and W_ext_ is lower in P compared with N steps, in all three impairments.

Several other articles confirmed that in unilateral impairments, as a rule, less muscular work is provided by the affected lower limb. For instance, this is the case for anterior cruciate ligament reconstruction (Hart et al., 2010), and above-knee amputation (Kowal et al., 2018). In the case of stroke, it has been shown that this asymmetry may go beyond the one required by the intrinsic muscle weakness, thus revealing a central “sparing” of the affected leg (Milot et al., 2007).

A normal W_ext,m_ was also found in patients with multiple sclerosis who are assumed to walk rather symmetrically, although this is not specified in the cited article (Wurdeman et al., 2013). Previous studies evidenced that MS patients suffer from an increased metabolic cost of walking, compared to healthy subjects walking at the same velocity (Olgiati et al., 1988). This extra cost, however, could be mostly ascribed to agonist-antagonist coactivation in spasticity, which does not generate mechanical work.

One unique study on healthy subjects walking on a treadmill contradicts findings from patients (Ellis et al., 2013). Healthy subjects were asked to adopt asymmetric step periods with the help of specific visual feedback. Compared with symmetric walking at the same velocity, asymmetric walking implied an increase in both metabolic expenditure and W_ext_ per unit time. This isolated, yet robust, observation has at least two explanations. First, unlike for healthy subjects, the primary aim of patients may be to minimize the muscle power provided by the impaired lower limb, thus creating a different, and paradoxically efficient, overall motor strategy. Second, the method used to calculate W_ext,step_ likely provides overestimation compared to the double-integration method (see Supplementary Note S2).

Taken together, the above findings explain the paradox of the observation of normal cost in many asymmetric patients: the affected lower limb is underloaded while the unaffected, or, less affected, lower limb is overloaded, just by an amount leading to an overall normal energy expenditure per unit distance covered.

Cavagna, G. A., Tesio, L., Fuchimoto, T., and Heglund, N. C. (1983). Ergometric evaluation of pathological gait. *J. Appl. Physiol.* 55, 607–613. doi:10.1152/jappl.1983.55.2.606.

Darcy S. Reisman, Katherine S. Rudolph, and William B. Farquhar (2009). Influence of speed on walking economy poststroke. *Neurorehabil. Neural Repair* 23, 529–534. doi:10.1177/1545968308328732.

Ellis, R. G., Howard, K. C., and Kram, R. (2013). The metabolic and mechanical costs of step time asymmetry in walking. *Proc. R. Soc. B Biol. Sci.* 280. doi:10.1098/rspb.2012.2784.

Hart, J. M., Ko, J. W. K., Konold, T., and Pietrosimione, B. (2010). Sagittal plane knee joint moments following anterior cruciate ligament injury and reconstruction: A systematic review. *Clin. Biomech.* 25, 277–283. doi:10.1016/j.clinbiomech.2009.12.004.

Kowal, M., Paprockza Borowicz, M., Starczewska, A., and Rutkowska-Kucharska, A. (2018). Biomechanical parameters of gait after unilateral above-knee amputation. Current state of research. *Ortop Traumatol Rehabil* 20, 245–256. doi:10.5604/01.3001.0012.3355.

Mahon, C. E., Darter, B. J., Dearth, C. L., and Hendershot, B. D. (2019). The relationship between gait symmetry and metabolic demand in individuals with unilateral transfemoral amputation: a preliminary study. *Mil. Med.* 00, 1–7. doi:10.1093/milmed/usy424.

Margaria, R. (1938). *Sulla fisiologia e specialmente sul consumo energetico della marcia e della corsa a varie velocità ed inclinazioni del terreno Accademia nazionale dei Lincei, Memorie*. Bardi, Roma.

Milot, M.-H., Nadeau, S., and Gravel, D. (2007). Muscular utilization of the plantarflexors, hip flexors and extensors in persons with hemiparesis walking at self-selected and maximal speeds. *J. Electromyogr. Kinesiol.* 17, 184–193. doi:10.1016/j.jelekin.2006.01.001.

Olgiati, R., Burgunder, J., and Mumenthaler, M. (1988). Increased energy cost of walking in multiple sclerosis: effect of spasticity, ataxia, and weakness. *Arch. Phys. Med. Rehabil.* 69, 846–849.

Roemmich, R. T., Leech, K. A., Gonzalez, A., and Bastian, A. (2019). Trading symmetry for energy cost during walking in healthy adults and persons poststroke. *Neurorehabil. Neural Repair*.

Rota, V., Benedetti, M. G., Okita, Y., Manfrini, M., and Tesio, L. (2016). Knee rotationplasty: motion of the body centre of mass during walking. *Int. J. Rehabil. Res.* 39, 346–353. doi:10.1097/MRR.0000000000000195.

Tesio, L. (1991). From neuroplastic potential to actual recovery after stroke: a call for cooperation between drugs and exercise. *Aging (Albany. NY).* 3, 97–8.

Tesio, L., Civaschi, P., and Tessari, L. (1985). Motion of the center of gravity of the body in clinical evaluation of gait. *Am. J. Phys. Med.* 64, 57–70.

Tesio, L., Lanzi, D., and Detrembleur, C. (1998). The 3-D motion of the centre of gravity of the human body during level walking. II. Lower limb amputees. *Clin. Biomech.* 13, 83–90. doi:10.1016/S0268-0033(97)00081-8.

Tesio, L., Rota, V., and Perucca, L. (2011). The 3D trajectory of the body centre of mass during adult human walking: Evidence for a speed-curvature power law. *J. Biomech.* 44, 732–740. doi:10.1016/j.jbiomech.2010.10.035.

Waters, R. L., and Mulroy, S. (1999). The energy expenditure of normal and pathologic gait. *Gait Posture* 9, 207–231.

Wurdeman, S. R., Huisinga, J. M., Filipi, M., and Stergiou, N. (2013). Multiple sclerosis alters the mechanical work performed on the body’s center of mass during gait. *J. Appl. Biomech.* 29, 435–42.

Zamparo, P., Francescato, M., De Luca, G., Lovati, L., and di Prampero, P. (1995). The energy cost of level walking in patients with hemiplegia. *Scand J Med Sci Sport.* 5, 348–352.
