# Supplementary material for: The Motion of Body Center of Mass During Walking: A Review Oriented to Clinical Applications
Source: Front Neurol. 2019 Sep 20;10:999. doi: 10.3389/fneur.2019.00999 (PMC6763727; doi:10.3389/fneur.2019.00999)
Supplement: Supplementary file 6 [file Table_6.docx]

**Note S6. “Seeing” the motion of the CoM during walking**

The CoM is a virtual point unbounded to any anatomical landmark. Perhaps for these reasons alterations of the CoM did not attract clinical research. On the other hand, optimizing the CoM trajectory in terms of energy expenditure and maintenance of balance can be thought of as one of the main goals of segmental motions, hence, of the neural control of walking. Can the alterations of this trajectory be at least suspected from some segmental motions detectable by the naked eye, without complex instrumental recordings? The answer is yes. Here are some hints taken from the authors’ experience.

Symmetric gaits

a) A slow velocity (Tesio, 1991) and, within any given velocity, steps shorter than normal (Cavagna and Franzetti, 1986), imply inefficiency in the transfer between kinetic and gravitational potential energy, which is constrained by the physical law of the pendulum. The cost of gait per unit distance increases. This increase is a good reason to investigate the causes for this alteration of the system mechanics.

b) A delayed or limited plantar flexion usually reflects balance difficulties. This phenomenon can be reliably detected by skilled clinicians (McGinley et al., 2006). The plantar flexors are the main engine of walking. When the corresponding foot is in the rear position, these muscles generate the “push-off” phase, by enhancing the forward acceleration of the CoM (Meinders et al., 1998; Liu et al., 2006; Honeine et al., 2013). Therefore walking with “flat” feet or, at the extreme, with feet “shuffling” on the ground (Merello et al., 2010), reveals an attempt to hold a more uniform velocity of the CoM, both in the sagittal and the frontal plane. Balance control is facilitated (Mazzà et al., 2008)(MacKinnon and Winter, 1993).

c) A crouched posture often reveals balance deficits. It implies lowering the CoM, thus making more stable the whole system. This posture also facilitates the visual anchoring to the ground. The contracted hip and knee extensors provide enhanced proprioceptive feedback (Tesio et al., 2017).

Asymmetric gaits (claudication)

A shorter “posterior” step of one lower limb reveals lower work on the CoM by that limb. Most of this work is done during the push-off phase. A shorter duration of one step, generating a visible “escape” limp, reveals a lower external work provided by the rear limb. This relationship does not hold during split-belt walking when step duration is constrained by the faster belt (see main text). Both spatial and temporal step asymmetries may imply an overall normal external work along with the whole stride, hence per unit distance (Tesio, 1991). This adaptation may work against rehabilitation attempts to bring back walking to symmetry (Rota et al., 2016; Tesio et al., 2018) (see also main text).

Cavagna, G. A., and Franzetti, P. (1986). The determinants of the step frequency in walking in humans. *J. Physiol.* 373, 235–242. doi:10.1113/jphysiol.1986.sp016044.

Honeine, J. L., Schieppati, M., Gagey, O., and Do, M. C. (2013). The functional role of the triceps surae muscle during human locomotion. *PLoS One* 8. doi:10.1371/journal.pone.0052943.

Liu, M. Q., Anderson, F. C., Pandy, M. G., and Delp, S. L. (2006). Muscles that support the body also modulate forward progression during walking. 39, 2623–2630. doi:10.1016/j.jbiomech.2005.08.017.

MacKinnon, C. D., and Winter, D. A. (1993). Control of whole body balance in the frontal plane during human walking. *J. Biomech.* 26, 633–44. doi:10.1016/0021-9290(93)90027-C.

Mazzà, C., Iosa, M., Pecoraro, F., Cappozzo, A., Saunders, J., Inman, V., et al. (2008). Control of the upper body accelerations in young and elderly women during level walking. *J. Neuroeng. Rehabil.* 5, 30. doi:10.1186/1743-0003-5-30.

McGinley, J. L., Morris, M. E., Greenwood, K. M., Goldie, P. A., and Olney, S. J. (2006). Accuracy of clinical observations of push-off during gait after stroke. *Arch. Phys. Med. Rehabil.* 87, 779–785. doi:10.1016/j.apmr.2006.02.022.

Meinders, M., Gitter, A., and Czerniecki, J. M. (1998). The role of ankle plantar flexor muscle work during walking. *Scand. J. Rehabil. Med.* 30, 39–46. doi:10.1080/003655098444309.

Merello, M., Fantacone, N., and Balej, J. (2010). Kinematic study of whole body center of mass position during gait in Parkinson’s disease patients with and without festination. *Mov. Disord.* 25, 747–754. doi:10.1002/mds.22958.

Rota, V., Benedetti, M. G., Okita, Y., Manfrini, M., and Tesio, L. (2016). Knee rotationplasty: motion of the body centre of mass during walking. *Int. J. Rehabil. Res.* 39, 346–353. doi:10.1097/MRR.0000000000000195.

Tesio, L. (1991). From neuroplastic potential to actual recovery after stroke: a call for cooperation between drugs and exercise. *Aging (Albany. NY).* 3, 97–8.

Tesio, L., Malloggi, C., Malfitano, C., Coccetta, C. A., Catino, L., and Rota, V. (2018). Limping on split-belt treadmills implies opposite kinematic and dynamic lower limb asymmetries. *Int. J. Rehabil. Res.* 41, 304–315. doi:10.1097/MRR.0000000000000320.

Tesio, L., Rota, V., Malloggi, C., Brugliera, L., and Catino, L. (2017). Crouch gait can be an effective form of forced-use/no constraint exercise for the paretic lower limb in stroke. *Int. J. Rehabil. Res.* 40, 254–267. doi:10.1097/MRR.0000000000000236.
