# Supplementary material for: The Motion of Body Center of Mass During Walking: A Review Oriented to Clinical Applications
Source: Front Neurol. 2019 Sep 20;10:999. doi: 10.3389/fneur.2019.00999 (PMC6763727; doi:10.3389/fneur.2019.00999)
Supplement: Supplementary file 7 [file Table_7.docx]

**Note S7. Split-belt walking as a forced-use rehabilitation paradigm**

S7.1. Rationale for split-belt-based treatments

In split-belt walking the subject walks on a force treadmill constructed with two parallel independent belts that can rotate at different velocities, hence the terms split- or dual-belt treadmill. The velocity ratio between the belts can range from 1.5 to 4, depending on the study. Splitting causes a sudden escape limp in healthy subjects; within a few strides, stance time and “anterior” step length are reduced on the faster belt. In therapeutic applications, the patient can be positioned with the affected leg—the one “escaping” during ground walking—on the slower belt, thus forcing protraction of the stance phase of this leg. However, this configuration enhances spatial asymmetry as the anterior step becomes longer on the impaired side. Most previous studies have focused on the spatial asymmetry and the error-augmentation pedagogic principle (Kao et al., 2013), which states that placement of the paretic limb on the slower belt will temporarily enhance spatial asymmetry (Helm and Reisman, 2015). An after-effect that reverses, or at least attenuates, the original asymmetry develops when ground walking is restored, reflecting the acquisition of novel inter-limb coordination (Lauzière et al., 2014).

S7.2. Split-belt walking in healthy subjects is not equivalent to real pathologic claudication.

Some basic issues concerning split-belt training remain controversial. Initial studies were based on kinematic observations, only, and focused on restoration of step-length asymmetry. The analogy between experimental claudication in healthy subjects and true pathological claudication during ground walking is valid for stance duration, which is shorter for the faster leg of healthy subjects on the treadmill and for the paretic leg of patients during ground walking. However, this is not valid for step length. In healthy subjects, the leg on the faster belt is pulled back meaning that the anterior step gets shorter, which does not reflect the paretic step of most patients with unilateral impairment during ground walking. Most importantly, dynamic asymmetries that are induced in healthy subjects by the split belt are opposite to those observed in hemiparetic subjects during ground walking. In healthy subjects, the faster leg is forced to provide a higher peak power than the slower leg due to heavy overloading of the plantar flexors during the push-off phase (Fig. S7-1), thus avoiding being dragged backwards by the


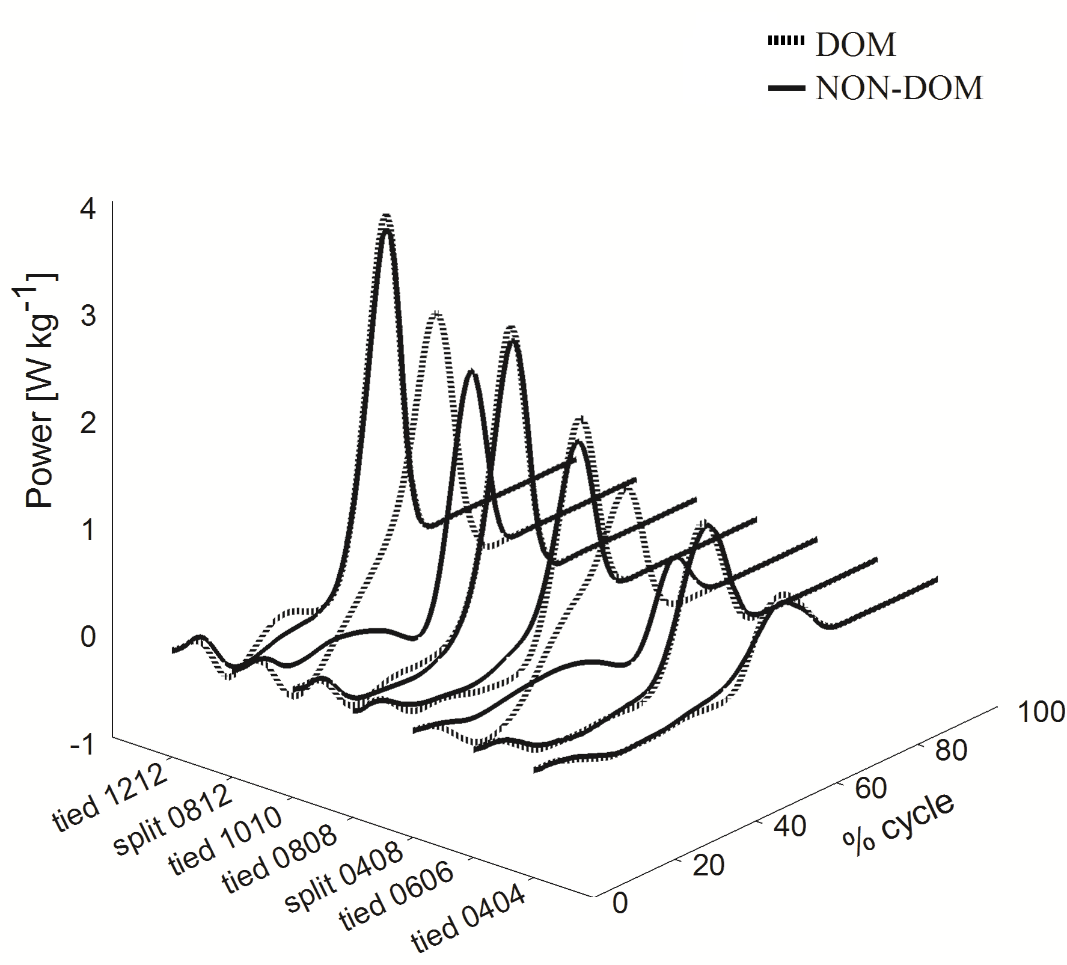


Figure S7-1: Walking was analyzed on a split-belt, force-sensing treadmill. Graph of weight-normalized ankle power (positive values for plantar flexion) in the sagittal plane (ordinate) as a function of both the standardized stride time (right abscissa) and the belt speed combinations (tied or split-belt conditions, left abscissa). Data were grand-averaged across 10 healthy adults (five men) and six strides per participant. In each category of the left abscissa, the first and the second pair of digits give the velocity of the belts assigned to the non-dominant (NON-DOM, hatched lines) and dominant side (DOM, continuous lines), respectively (for example, 0408 indicates velocities of 0.4 and 0.8 m s^-1^, respectively). Higher peak power is generated by the plantar flexors on the faster belt compared with the slower belt. This peak is lower, for any given speed, when walking occurs in a split- rather than on a tied-belt modality (compare curves 1212 and 0812). In contrast, the peak is the same when the velocity of the faster leg in the split-belt modality is compared with the average velocity in the tied-belt modality (for example, curve 0812 vs. 1010). This form of representation demonstrates that a greater amount of power is absorbed at the ankle in late stance on the faster belt and then generated earlier and for a longer relative duration hence, much more positive work is provided by muscles compared with the slow belt. Therefore, the split-belt arrangement induces an original form of body propulsion, exploiting the energy storage of the plantar flexors, allowed by the dragging (faster) belt. Taken from Tesio et al. (2018), used with permission.

faster belt. In addition, the time course of power absorption and production is altered on the faster side (further explained in the legend of Fig. S7-1).

In contrast, the paretic limb of subjects with hemiparesis usually exhibits a longer anterior step compared with the non-paretic leg during ground walking, and also produces less power at push off (Reisman et al., 2009; Finley et al., 2015; Malone and Bastian, 2014; Tesio et al., 2018). This leads to the question of whether we should aim to achieve kinematic or dynamic symmetry. Forcing power production from the paretic lower limb by placing it on the faster belt seems an attractive option; however, creating more symmetric power production during exercise can lead to the after-effect of asymmetric ankle power production in the long term (see also main text) (Lauzière et al., 2016). To further weaken the resemblance to pathologic claudication, adaptation develops after 10 to 20 minutes of split-belt walking; kinematic asymmetries become less pronounced and the whole-body metabolic expenditure decreases (Finley et al., 2013). It has been suggested (Sánchez et al., 2019) that subjects adapt to take advantage of the energy input from the faster belt (which runs faster than the CoM which is traveling at an intermediate velocity between that of the two belts). This may explain both the slow decrease in work of the plantar flexors on the faster belt and the paradoxical decrease in overall energy expenditure per unit distance. In summary, optimal design of the exercise session in terms of the choice of belt assigned to the paretic limb, timing and sequencing of walking bouts and session schedules are still unclear.

Finley, J. M., Bastian, A. J., and Gottschall, J. S. (2013). Learning to be economical: The energy cost of walking tracks motor adaptation. *J. Physiol.* 591, 1081–1095. doi:10.1113/jphysiol.2012.245506.

Finley, J. M., Long, A., Bastian, A. J., and Torres-Oviedo, G. (2015). Spatial and temporal control contribute to step length asymmetry during split-belt adaptation and hemiparetic gait. *Neurorehabil. Neural Repair* 29, 786–795. doi:10.1177/1545968314567149.

Helm, E. E., and Reisman, D. S. (2015). The split-belt walking paradigm: exploring motor learning and spatiotemporal asymmetry poststroke. *Phys. Med. Rehabil. Clin. N. Am.* 26, 703–713. doi:10.1016/j.pmr.2015.06.010.

Kao, P.-C., Srivastava, S., Agrawal, S., and Scholz, J. (2013). Effect of robotic performance-based error-augmentation versus error-reduction training on the gait of healthy individuals. *Gait Posture* 71, 233–236. doi:10.1038/mp.2011.182.doi.

Lauzière, S., Mièville, C., Betschart, M., Duclos, C., Aissaoui, R., and Nadeau, S. (2014). Plantarflexion moment is a contributor to step length after-effect following walking on a split-belt treadmill in individuals with stroke and healthy individuals. *J. Rehabil. Med.* 46, 849–857. doi:10.2340/16501977-1845.

Lauzière, S., Mièville, C., Betschart, M., Duclos, C., Aissaoui, R., and Nadeau, S. (2016). A more symmetrical gait after split-belt treadmill walking increases the effort in paretic plantar flexors in people post-stroke. *J. Rehabil. Med.* 48, 576–582. doi:10.2340/16501977-2117.

Malone, L. A., and Bastian, A. J. (2014). Spatial and temporal asymmetries in gait predict split-belt adaptation behavior in stroke. *Neurorehabil. Neural Repair* 28, 230–240. doi:10.1177/1545968313505912.

Reisman, D. S., Wityk, R., Silver, K., and Bastian, A. J. (2009). Split-belt treadmill adaptation transfers to overground walking in persons poststroke. *Neurorehabil. Neural Repair* 23, 735–44. doi:10.1177/1545968309332880.

Sánchez, N., Simha, S. N., Donelan, J. M., James, M., and Finley, J. M. (2019). Taking advantage of external mechanical work to reduce metabolic cost: the mechanics and energetics of split-belt treadmill walking. *J. Physiol.*, 1–44. doi:doi.org/10.1101/500835.

Tesio, L., Malloggi, C., Malfitano, C., Coccetta, C. A., Catino, L., and Rota, V. (2018). Limping on split-belt treadmills implies opposite kinematic and dynamic lower limb asymmetries. *Int. J. Rehabil. Res.* 41, 304–315. doi:10.1097/MRR.0000000000000320.

References

Finley, J. M., Bastian, A. J., and Gottschall, J. S. (2013). Learning to be economical: The energy cost of walking tracks motor adaptation. *J. Physiol.* 591, 1081–1095. doi:10.1113/jphysiol.2012.245506.

Finley, J. M., Long, A., Bastian, A. J., and Torres-Oviedo, G. (2015). Spatial and temporal control contribute to step length asymmetry during split-belt adaptation and hemiparetic gait. *Neurorehabil. Neural Repair* 29, 786–795. doi:10.1177/1545968314567149.

Helm, E. E., and Reisman, D. S. (2015). The split-belt walking paradigm: exploring motor learning and spatiotemporal asymmetry poststroke. *Phys. Med. Rehabil. Clin. N. Am.* 26, 703–713. doi:10.1016/j.pmr.2015.06.010.

Kao, P.-C., Srivastava, S., Agrawal, S., and Scholz, J. (2013). Effect of robotic performance-based error-augmentation versus error-reduction training on the gait of healthy individuals. *Gait Posture* 71, 233–236. doi:10.1038/mp.2011.182.doi.

Lauzière, S., Mièville, C., Betschart, M., Duclos, C., Aissaoui, R., and Nadeau, S. (2014). Plantarflexion moment is a contributor to step length after-effect following walking on a split-belt treadmill in individuals with stroke and healthy individuals. *J. Rehabil. Med.* 46, 849–857. doi:10.2340/16501977-1845.

Lauzière, S., Mièville, C., Betschart, M., Duclos, C., Aissaoui, R., and Nadeau, S. (2016). A more symmetrical gait after split-belt treadmill walking increases the effort in paretic plantar flexors in people post-stroke. *J. Rehabil. Med.* 48, 576–582. doi:10.2340/16501977-2117.

Malone, L. A., and Bastian, A. J. (2014). Spatial and temporal asymmetries in gait predict split-belt adaptation behavior in stroke. *Neurorehabil. Neural Repair* 28, 230–240. doi:10.1177/1545968313505912.

Reisman, D. S., Wityk, R., Silver, K., and Bastian, A. J. (2009). Split-belt treadmill adaptation transfers to overground walking in persons poststroke. *Neurorehabil. Neural Repair* 23, 735–44. doi:10.1177/1545968309332880.

Sánchez, N., Simha, S. N., Donelan, J. M., James, M., and Finley, J. M. (2019). Taking advantage of external mechanical work to reduce metabolic cost: the mechanics and energetics of split-belt treadmill walking. *J. Physiol.*, 1–44. doi:doi.org/10.1101/500835.

Tesio, L., Malloggi, C., Malfitano, C., Coccetta, C. A., Catino, L., and Rota, V. (2018). Limping on split-belt treadmills implies opposite kinematic and dynamic lower limb asymmetries. *Int. J. Rehabil. Res.* 41, 304–315. doi:10.1097/MRR.0000000000000320.

Finley, J. M., Bastian, A. J., and Gottschall, J. S. (2013). Learning to be economical: The energy cost of walking tracks motor adaptation. *J. Physiol.* 591, 1081–1095. doi:10.1113/jphysiol.2012.245506.

Finley, J. M., Long, A., Bastian, A. J., and Torres-Oviedo, G. (2015). Spatial and temporal control contribute to step length asymmetry during split-belt adaptation and hemiparetic gait. *Neurorehabil. Neural Repair* 29, 786–795. doi:10.1177/1545968314567149.

Helm, E. E., and Reisman, D. S. (2015). The split-belt walking paradigm: exploring motor learning and spatiotemporal asymmetry poststroke. *Phys. Med. Rehabil. Clin. N. Am.* 26, 703–713. doi:10.1016/j.pmr.2015.06.010.

Kao, P.-C., Srivastava, S., Agrawal, S., and Scholz, J. (2013). Effect of robotic performance-based error-augmentation versus error-reduction training on the gait of healthy individuals. *Gait Posture* 71, 233–236. doi:10.1038/mp.2011.182.doi.

Lauzière, S., Mièville, C., Betschart, M., Duclos, C., Aissaoui, R., and Nadeau, S. (2014). Plantarflexion moment is a contributor to step length after-effect following walking on a split-belt treadmill in individuals with stroke and healthy individuals. *J. Rehabil. Med.* 46, 849–857. doi:10.2340/16501977-1845.

Lauzière, S., Mièville, C., Betschart, M., Duclos, C., Aissaoui, R., and Nadeau, S. (2016). A more symmetrical gait after split-belt treadmill walking increases the effort in paretic plantar flexors in people post-stroke. *J. Rehabil. Med.* 48, 576–582. doi:10.2340/16501977-2117.

Malone, L. A., and Bastian, A. J. (2014). Spatial and temporal asymmetries in gait predict split-belt adaptation behavior in stroke. *Neurorehabil. Neural Repair* 28, 230–240. doi:10.1177/1545968313505912.

Reisman, D. S., Wityk, R., Silver, K., and Bastian, A. J. (2009). Split-belt treadmill adaptation transfers to overground walking in persons poststroke. *Neurorehabil. Neural Repair* 23, 735–44. doi:10.1177/1545968309332880.

Sánchez, N., Simha, S. N., Donelan, J. M., James, M., and Finley, J. M. (2019). Taking advantage of external mechanical work to reduce metabolic cost: the mechanics and energetics of split-belt treadmill walking. *J. Physiol.*, 1–44. doi:doi.org/10.1101/500835.

Tesio, L., Malloggi, C., Malfitano, C., Coccetta, C. A., Catino, L., and Rota, V. (2018). Limping on split-belt treadmills implies opposite kinematic and dynamic lower limb asymmetries. *Int. J. Rehabil. Res.* 41, 304–315. doi:10.1097/MRR.0000000000000320.
